# An Overview of Recent Developments in the Management of Burn Injuries

**DOI:** 10.3390/ijms242216357

**Published:** 2023-11-15

**Authors:** Elżbieta Radzikowska-Büchner, Inga Łopuszyńska, Wojciech Flieger, Michał Tobiasz, Ryszard Maciejewski, Jolanta Flieger

**Affiliations:** 1 Department of Plastic, Reconstructive and Maxillary Surgery, National Medical Institute of the Ministry of the Interior and Administration, Wołoska 137 Street, 02-507 Warszawa, Poland; ingalopuszynska@gmail.com; 2 Department of Human Anatomy, Medical University of Lublin, Jaczewskiego 4 Street, 20-090 Lublin, Poland; wwoj24@wp.pl; 3 Department of Plastic Surgery, Reconstructive Surgery and Burn Treatment, Medical University of Lublin, Krasnystawska 52 Street, 21-010 Łęczna, Poland; misq2@onet.pl; 4 Faculty of Medicine, University of Warsaw, Żwirki i Wigury 101 Street, 02-089 Warszawa, Poland; maciejewski.r@gmail.com; 5 Department of Analytical Chemistry, Medical University of Lublin, Chodźki 4A Street, 20-093 Lublin, Poland

**Keywords:** burn injuries, burn care, regenerative medicine, skin grafts, skin tissue engineering, dressing

## Abstract

According to the World Health Organization (WHO), around 11 million people suffer from burns every year, and 180,000 die from them. A burn is a condition in which heat, chemical substances, an electrical current or other factors cause tissue damage. Burns mainly affect the skin, but can also affect deeper tissues such as bones or muscles. When burned, the skin loses its main functions, such as protection from the external environment, pathogens, evaporation and heat loss. Depending on the stage of the burn, the patient’s condition and the cause of the burn, we need to choose the most appropriate treatment. Personalization and multidisciplinary collaboration are key to the successful management of burn patients. In this comprehensive review, we have collected and discussed the available treatment options, focusing on recent advances in topical treatments, wound cleansing, dressings, skin grafting, nutrition, pain and scar tissue management.

## 1. Introduction

Burns are among the most serious and painful of injuries, often affecting children, people with disabilities and the elderly, whose numbers are increasing in ageing societies [1,2,3,4,5,6,7]. Yakupu et al. [8] collected annual case data on burn incidence, deaths and disability-adjusted life years (DALYs) and analyzed international burn trends in 204 countries from 1990 to 2019. The data were obtained from the Institute for Health Metrics and Evaluation and evaluated using the Global Health Data Exchange (GHDx) [9]. The authors identified 8,378,122 new cases of burns in 2019 alone, most of which were in the part of the population aged 10–19. Burns were the cause of death in 111,292 cases, mostly in children aged 1–4 years. At the national level, the highest number of burns were recorded in China, followed by India, in 2019. Based on the data collected, the authors observed a decrease in DALYs and mortality, and an increase in the number of new cases worldwide. The authors confirmed that burns are more common in younger age groups. Using the Socio-Demographic Index (SDI), they assessed the increased risk of burns with lower a SDI, high occupational risk and alcohol abuse.

Burns are the fourth most common type of injury after road traffic accidents, falls and physical violence [10]. With climate change and rising temperatures, sunburns, which are associated with a variety of skin cancers including melanoma, are also increasing [11]. Guy et al. reported in 2016 that, according to data from the US, the cost of sunburn treatment exceeds USD 11 million annually [12].

In recent decades, many burn treatment centers have been established, providing specialist care and helping to reduce mortality [13,14]. Although progress has been significant, the treatment of burns is perceived as burdensome due to numerous complications and a relatively long hospital stay. In addition, as the number of patients with severe burns increases, new issues arise, such as post-traumatic care, scar management and the management of psychological problems, as patients often present with post-traumatic stress disorder (PTSD) [5,15].

**Figure 1:**
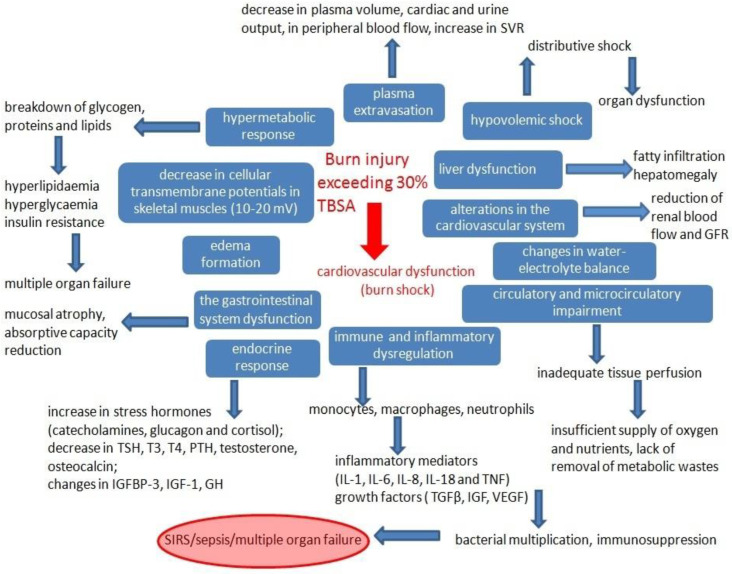
The systemic changes caused by burns that exceed 30% of TBSA. Abbreviations: glomerular filtration rate (GFR), systemic vascular resistance (SVR), systemic inflammatory response syndrome (SIRS), thyroid stimulating hormone (TSH), triiodothyronine (T3), thyroxine (T4), insulin-like growth factor binding protein-3 (IGFBP-3), transforming growth factor-β (TGF-β), insulin-like growth factor (IGF), insulin-like growth factor 1 (IGF-1), vascular endothelial growth factor (VEGF), tumor necrosis factor (TNF), growth hormone (GH), interleukin 1, 6, 8, 18 (IL-1, -6, -8, -18), and total body surface area (TBSA).

Burns on more than 30% of the total body surface area (TBSA) cause burn shock. The systemic changes that occur in response to thermal injury are shown schematically in Figure 1.

**Figure 2:**
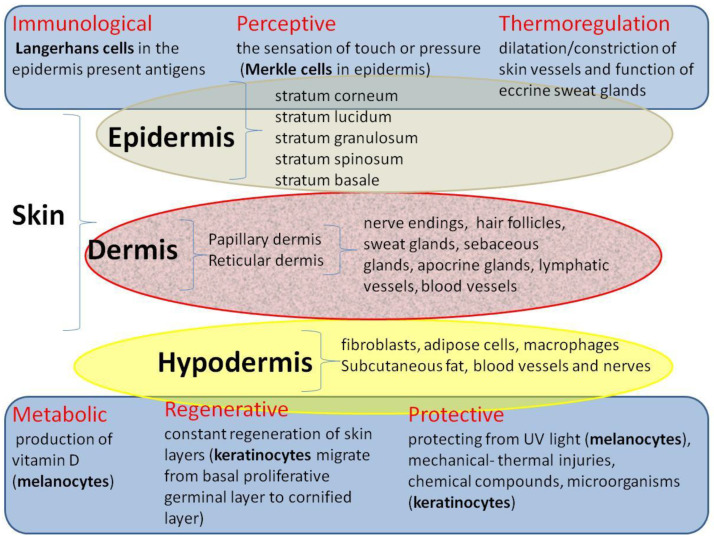
The physiological functions of the human skin.

The consequences of burns are extremely serious considering the fact that the skin is the largest organ in the human body, accounting for approximately 8% of total body weight. Human skin covers an area of 1.2 to 2.2 m^2^ and is 0.5 to 4.0 mm thick [16]. It consists of several layers: the outermost epidermis, the dermis (papillary and reticular) and the subcutaneous tissue, which perform different physiological functions (Figure 2).

**Figure 3:**
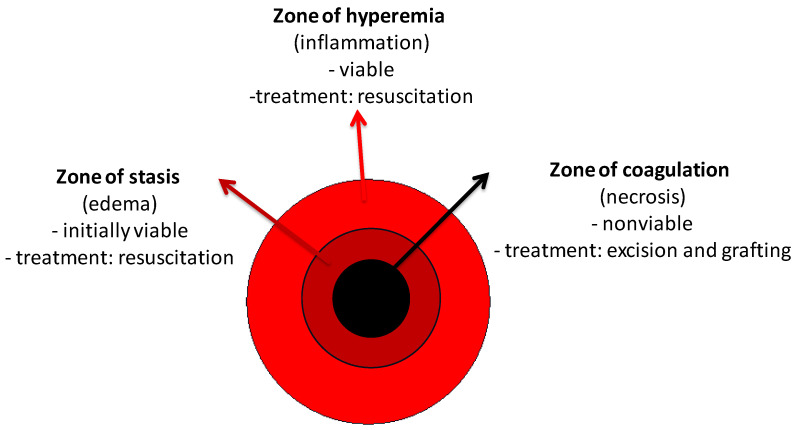
The areas of the skin that are at different distances from the damaging agent.

A burn is a condition in which the skin barrier is disrupted by external thermal, chemical, or electrical factors. When the integrity of the skin is altered, its functions are compromised and we are more susceptible to dehydration, infection, metabolic disorders, or even death. Histologically, the area of injury is divided into the three zones, described by Jackson in 1947 [17,18] (Figure 3). The skin has a high regenerative potential, but when it is injured beyond the reticular dermis, its regenerative capacity is reduced [19]. The process of wound healing involves three distinct phases: inflammation, which involves coagulation, cytokine release, chemotaxis, and cell recruitment; proliferation, which is the process of dermal resurfacing with phases of angiogenesis and fibroplasia; and finally, maturation, which involves the extracellular matrix [20,21]. Closure of the wound results in the formation of scar tissue. During this final stage of healing, there is a possibility of hypertrophic scarring, which causes contractions.

The healing time of burn wounds depends on their severity (superficial, superficial partial, deep partial, or complete wounds). While superficial burns covering the epidermis layer, although painful (second-degree), heal within a few weeks without leaving scars, a deep wound takes a long time to heal due to the destruction of the extracellular matrix (ECM) and the degradation of growth factors (GFs), a prolonged inflammatory phase, increases in pro-inflammatory cytokines, proteases, reactive oxygen species (ROS) and possible infection [22]. Burns can be classified into four main stages based on the depth of penetration, which depends on the exposure temperature, contact time, source of exposure and skin thickness [23,24,25]. Table 1 summarizes the classification of burns and the characteristics of each stage.

## 2. The Aim of the Review and Search Strategy

Treating burns is a challenge for medicine and requires a multidisciplinary approach. Treatment involves specialist nurses, clinicians with different specialties, biotechnologists, psychologists, physiotherapists, medical analysts, and pharmacists, who need to collaborate and communicate with each other. Most reviews are focused on a specific topic such as fluid resuscitation, nutrition, tissue engineering, dressing types, transplantation, etc. This narrative review collects studies that aim to comprehensively summarize the latest achievements in a wide range of topics including wound healing, escharotomy, preparation, excision, local treatment, different types of dressings, nutrition, pain management, and rehabilitation after burns. Attention was drawn to existing debates and research in the experimental phase. 

To identify current therapeutic strategies, an open search of the PubMed database was performed using the following terms: (burn therapy) OR (burn injury). After receiving 127,613 results from the years 1940 to 2023, we reviewed the articles for novelty in burn care, focusing on the last 10 years. The 48,137 articles identified were selected based on their title and abstract. Subsequent searches were performed in relation to the planned subchapters. The search included the following string: (injury OR treatment) AND (burns) AND (escharotomy OR debridement OR topical treatment OR dressing OR excision OR skin substitutes OR autografts OR skin grafts OR cultured epithelial autograft OR xenografts OR allografts OR human amnion OR artificial skin OR maggot debridement therapy OR negative pressure wound therapy OR hyperbaric oxygen therapy OR fish skin grafts OR nutrition OR pain management OR psychological advisory OR rehabilitation OR scar treatment OR antibiotic therapy).

The review included reviews, articles, randomized controlled trials, case reports, and clinical trials written in English. Articles were included if we had access to the full text, which allowed us to access older articles that were relevant to the issue under discussion. Repetitions were excluded. Eligibility for inclusion/exclusion was assessed individually by the senior authors, taking into account the aspects of novelty and relevance to the treatment of burns. A total of 468 articles were included in the review, which were qualitatively sorted by topic.

## 3. Initial Assessment

### 3.1. The Total Body Surface Area (TBSA)

**Figure 4:**
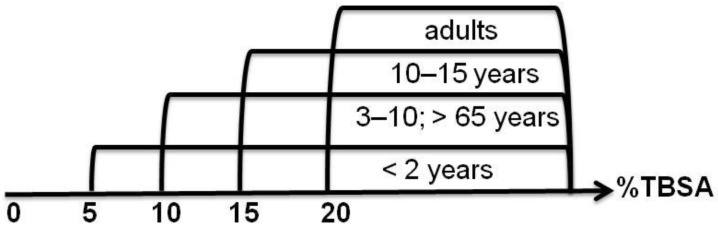
Selected criteria to support the decision to admit a patient to a specialized burn unit (age and %TBSA).

The estimated total body surface area and the estimated burn size may vary according to the applied estimation method (e.g., rule of nine, rule of palms, Lund-Browder-Chart), which can impact the initial management of burn patients. In practice, the extent of burns is estimated as a percent, using the so-called rule of nine [10,14], which assumes that individual body parts represent 9% (or a multiple thereof) of the total body surface area, i.e., each arm is 9%, each leg 18%, the torso 36% (18% back, 18% front), the head 9% and the genitals 1%, in adults. The figures are slightly different in children, where the head and neck occupy a larger proportion of the body [16,26]. Another useful rule for assessing the extent of a burn is to compare its surface area with the surface area of the patient’s hand, which is 1% of the total body surface area (TBSA) [23]. The decision to admit a patient is always made by a specialist in burn treatment centers, which have different levels of quality and procedures in different countries. Thanks to the work of doctors around the world, Clinical Practice Guidelines (CPGs) with uniform recommendations are prepared and continuously updated and expanded by different burns associations, such as the European Burns Association (EBA) [27] and American Burn Association (ABA) [28]. According to the guidelines, some of the important criteria for hospitalization are age and %TBSA [29] (Figure 4).

In addition to age, there are many other indications for hospitalization, such as the need for resuscitation due to burn shock, burns to the face, hands, genitals, or large joints, deep burns, peripheral burns, burns with associated injuries or diseases that may complicate treatment, prolonged recovery or mortality, burns with suspected respiratory injury, burns requiring special social and emotional support or long-term rehabilitation, and severe electrical and chemical burns. Patients with toxic epidermal necrolysis, necrotizing fasciitis, staphylococcal baby burn syndrome, etc., should also be referred to specialist burn centers. Hospitalization is always necessary if there is any doubt about treatment, even if only 10% of the skin is affected in children and the elderly, and 15% in adults.

### 3.2. Fluid Resuscitation

At the start of therapy, the patient’s condition should be stabilized, i.e., control of the airway, breathing, and circulation should be ensured [10], intravenous access established, and fluid resuscitation started. Only when the patient is stable can wound management and rehabilitation/reconstruction be started. The injury results in increased vascular permeability, which means the excessive leakage of plasma into the tissues, resulting in decreased serum volume, decreased cardiac output, decreased urine output and decreased peripheral blood flow [30]. This cascade of events is termed burn shock [18], characterized by hypovolemia and the release of various inflammatory markers, i.e., histamine and cytokines [31]. Burn shock occurs when the burnt TBSA is >20% (>15% in children) [14,18]. Prompt fluid resuscitation is essential to reduce post-traumatic shock, reduce burn depth, and reduce mortality [32]. Fluid administration is recommended for adults with a TBSA burn greater than 20% of their TBSA, and greater than 10% in children, and should be discontinued in patients without signs of hypovolemia, as it may exacerbate the formation of edema [33,34]. Excessive fluid overload is also dangerous because it can cause pulmonary and cerebral edema or compartment syndrome in the extremities, abdominal development of acute kidney injury (AKI) and even increases mortality. When exceeding 250 mL/kg/24 h, the so-called Ivy index increases the risk of abdominal compartment syndrome. Moreover, it has been shown that if the volume of fluids administered on the first day is larger, the need for fluids will also increase in the following days [35]. During fluid resuscitation, urine output (UOP) should be measured and should be greater than 0.5 mL/kg per hour, or 1 mL/kg per hour in patients with renal impairment and for children under 30 kg [36]. Base deficit (<2), lactic acid, blood pressure, and peripheral pulse should also be monitored. At the end of the 20th century, it had already been noticed that cardiac output facilitates the control of fluid therapy, but requires the placement of a catheter into the pulmonary artery [37]. Other methods, such as transpulmonary thermodilution and arterial pressure wave analysis, are less well-known. According to the recommendations of the American Burn Association (ABA), when their TBSA burn is <20%, patients can be rehydrated with oral solutions that contain pure water, glucose and electrolytes.

In patients with burns > 20%, intravenous resuscitation is performed using isotonic lactated Ringer’s solution with a sodium concentration of 130 mEq/L as the primary crystalloid, but a solution of Isolyte/Plasmalyte may also be used instead [38]. The volume of Ringer’s lactate to be administered in mL per day is calculated using the Parkland formula (the Baxter formula) of 4 mL × body weight (kg) × %TBSA.

Other sources provide wider ranges of total fluid requirements in ml per day from 3.7–4.3 mL/kg/%TBSA for adults to 3 mL/kg/%TBSA for children during the first 24 h [33], or from 4 to 8 mL × body weight (kg) × %TBSA (total body surface area) [39]. In essence, 50% of the calculated fluid requirement should be administered within the first 8 h and the remaining 50% within the next 16 h [1,10]. There are also other methods of calculating fluid volume, i.e., Brooke’s rule: 2 mL × body weight (kg) × %TBSA, or the tens rule: 10 mL/h × %TBSA.

Parkland’s formula appears to be controversial, mainly regarding its accuracy [40]. According to current research, intravenous resuscitation often leads to an overestimation of fluid administration (fluid creep). Therefore, a retrospective study was conducted on 480 resuscitated patients with burns greater than 19% of their TBSA, hospitalized over a 15-year period. Resuscitation was controlled according to UOP [41]. The study found no significant difference in complication rates (80 vs. 82%) or mortality (14 vs. 17%) between patients who received adequate or higher fluid volumes than those calculated using Parkland’s formula. 

A similar conclusion follows from the research of Daniels et al. [42], who confirmed that a restrictive fluid administration regime under Parkland’s supervision ensures better survival. The research was based on data from 569 adult patients with burns exceeding 20% of their TBSA. Patients who received an amount of fluid calculated using Parkland’s formula had a significantly lower mortality rate (4.5%) compared to the groups who received less (16.7%) or more (19.5%) (*p* = 0.021) in the first week. However, in the long term, higher survival (regression coefficients −0.11 and −0.086; odds ratio 0.896 and 0.918; 95% confidence interval (CI) 0.411–1.951 and 0.42–2.004) was observed in the groups that received lower amounts of fluids.

According to a prospective, non-interventional study conducted at 21 burn centers in the United States, the amount of fluid that is commonly administered in the first 24 h after the burn is sometimes greater than the estimated Parkland formula [43]. The study also determined that albumin supplementation should be initiated during resuscitation within the first 12 h after acute burns, especially in older age, larger and deeper burns, and in patients with the highest initial fluid requirements. The administration of 5% albumin or fresh frozen plasma (FFP) if the patient does not respond to crystalloid volume expansion is consistent with ABA recommendations. Albumin supplementation is able to improve the intake/output ratio (I/O) calculated by dividing the total hourly intake of fluid (mL/kg/%TBSA/h) by the urine output (UPO; mL/kg/h). An increase in the I/O ratio indicates a poor prognosis. The protocol implemented by the Army Burn Center as part of Operation Iraqi Freedom also includes the administration of colloids for initial treatment, as this reduces the incidence of abdominal compartment syndrome. It was noticed that in this case, the need for fluids for initial treatment increases almost twice as much as that calculated according to the Parkland formula [44].

Resuscitation is problematic in children who are more susceptible to hypothermia and have a greater need for fluids [45]. In addition to the Parkland formula, various approaches can be used to calculate fluid resuscitation in pediatric patients, i.e., The Lund and Browder chart, the Cincinnati formula, and the Galveston formula [46,47]. Due to the lower glycogen reserve in young children under 5 years of age, dextrose should be included in fluid resuscitation [45].

Despite existing guidelines for fluid resuscitation, over- and under-resuscitation are not uncommon in practice. Typically, recorded studies reveal that larger resuscitation volumes are administered in the first 24 h compared to the amount calculated using the Parkland formula [35,41,48,49].

Also, a recent retrospective study conducted at the Helsinki Burn Center in 2023, which included a population of 46 people after burn injury to 32 of their %TBSA, demonstrated the use of excessive fluid resuscitation. The median amount of fluid administered was 5.9 mL/kg/%TBSA/24 h [50]. 

Over-resuscitation can produce significant complications known as fluid creep. Therefore, the calculated volumes should instead be treated as a “starting point” for resuscitation. The volume of fluid administered should be adjusted to maintain urine output at approximately 0.5–1.0 mL/kg/h in adults and 1.0–1.5 mL/kg/h in children.

Although Ringer’s lactate is most often used for the resuscitation of burns, the alternative is Plasmalyte^®^ (PL), which is cheaper, which is not without significance because patients with large burns need a lot of fluids during resuscitation. Hypertonic lactate salt solution (HLS) with colloids can also be used for fluid resuscitation [39], however, colloids may penetrate the extravascular space and cause a change in oncotic pressure [51]. To date, guidelines and protocols have been established for fluid resuscitation on the first day after a burn to restore plasma volume in the intravascular compartment, but there is no consensus on the composition or type of fluid involved, namely, isotonic crystalloids, hypertonic solutions or colloids. The issue of finding an appropriate burn resuscitation fluid is still being explored [52].

There are several hypotheses regarding the causes of fluid overload during fluid resuscitation [53,54]. Firstly, the possibility of an incorrect assessment of the burnt %TBSA should be considered. This error is propagated when calculating the volume of resuscitation fluids. Saffle [55] suggests that the cause of fluid creep is excessive crystalloid administration combined with limited colloids, such as 20% albumin administration. There is speculation that goal-directed therapy (GDT) may lead to aggressive fluid resuscitation [56]. Another possibility is the so-called “opioid creep” [57], which results from the administration of high doses of opioids. In this case, peripheral vasodilation and a decrease in blood pressure somehow force an increase in the amount of fluid resuscitated. Prospective randomized trials [58] have shown beneficial effects in terms of reduced fluid requirements (3.0 vs. 5.5 mL kg^−1^ %TBSA^−1^, *p* < 0.01) and increased urine output, which may be aided by supplemental therapy with antioxidants such as vitamin C and glutathione. Retrospective studies have shown that in the early resuscitation period after therapeutic plasmapheresis, or permissive hypovolemia [59], reduced fluid intake and increased urine output are also observed [60]. To avoid “fluid creep”, it is advisable to use as little fluid as possible for resuscitation. Resuscitation should be individualized and monitored, for example, by measuring several parameters in addition to urine output (UO), such as intra-abdominal pressure (IAP) or pulse pressure variation (PPV), the global end diastolic volume index (GEDVI) or extravascular lung water index (EVLWI), which are evaluated by pulse contour analysis, and transpulmonary thermodilution.

### 3.3. Thermoregulation

The skin plays a key role in keeping the internal body temperature at a constant level, regardless of the ambient temperature. The thermoregulatory function of the skin is ensured by the regulation of blood perfusion through blood vessels and evaporation through sweat glands [61]. A deep and extensive burn, compromising the integrity of the skin, causes the loss of these abilities [62].

Some patients in the early phase of resuscitation develop a state of hypothermia within the first 72 h, which is induced by inflammatory mediators stimulated by the hypothalamic regulatory center. The condition worsens after procedures such as the administration of intravenous fluids without a warming system used during resuscitation, cooling and even the administration of general anesthesia [63]. Hypothermia in burn patients is indicated by an internal temperature lower than 36.5 °C, while for healthy people the threshold is 35 °C [64].

The mere fact of hypothermia occurring in the early phase after injury worsens the prognosis in terms of mortality and the duration of treatment, regardless of other clinical factors [64,65]. However, it has been noted that hypothermia occurs more often in older women and with burns exceeding 40% of their TBSA [66]. Researchers at the University of Pittsburgh’s Department of Emergency Medicine collected data from approximately 3000 burn patients admitted to a Pennsylvania emergency department over a 10-year period [67]. Hypothermia (<36.5 °C) occurred in 42% (1163) of patients. It was found that the risk of hypothermia increases by 62% in patients whose burns exceeded 40% of their TBSA, in patients over 51 years of age, in patients requiring intubation and in patients in a coma (Glasgow Coma Scale 8). Accumulated data also indicate that adjusted mortality increased in patients who were admitted to the burn unit with hypothermia. Another retrospective cross-sectional study was performed on 57 patients with burns involving 34.56% ± 16.64 of their TBSA [66]. Based on the data, a logistic regression model in the form of a ROC curve was developed. Hypothermia on admission occurred in 79.2% of patients, which was statistically significantly related to the extent of the burn (*p* = 0.003). In turn, when their average temperature did not exceed 36 °C for at least 16 h after the burn, the probability of death was significantly higher (*p* = 0.033).

In turn, the hypermetabolic reaction that occurs in the next phase after the burn results in hyperthermia [18,68]. The increase in resting energy expenditure may increase by up to approximately 80%, depending on the extent of the burn [69]. An unbalanced state of hyperthermia worsens the hypermetabolic response, which in the worst case will lead to multi-organ failure [70].

In 1975, a study by Wilmore et al. [71] observed that increasing the ambient temperature to 28–33 °C is one effective way to mitigate the hypermetabolic reaction.

The International Burn Society (ISBI) recommends maintaining a basal body temperature of at least 36 °C [72], but strategies for managing hypo- and hyperthermic patients are usually decided by local burn centers. In the UK, hypothermia was most often treated by increasing the ambient temperature, while hyperthermia was most often treated by administering paracetamol [73]. Most burn centers in the United States and Canada maintain an internal temperature of 36 to 38 °C in the operating room and an ambient temperature of approximately 24 to 35 °C in the intensive care unit. Most centers see the benefits of increased ambient temperature, but at the same time see a greater risk of the contamination of sterile fields and a deterioration in work comfort [74].

At present, more and more attention is being paid to the study of the mechanisms of the development of sweat glands, which are responsible for the thermoregulatory function in humans. In patients with deep burns, the glands are damaged, leading to a loss of thermoregulation. The identification of the cascade signaling pathways involved in sweat gland development can be used to reconstruct sweat adenoid cells. So far, the involvement of the following signaling pathways has been identified: Wnt/β-catenin, ectodysplasin A/ectodysplasin A receptor/nuclear factor κB, sonic hedgehog and forkhead box [75,76] transcription factor. Recently, attempts have been made to differentiate stem cells into a sweat gland-like tissue with a specific secretory function [76,77].

## 4. Wound Healing

To date, there is no widely used burn dressing that provides complete healing without multiple dressing changes, additional surgery and skin grafts [78]. The treatment process involves several stages, including conservative measures for burn shock and wound protection, followed by surgical procedures such as tissue excision and closure with skin grafts [79]. This approach is stressful for patients and does not guarantee successful healing or survival. Scientists are actively searching for the gold standard in burn care to improve and accelerate the healing process while minimizing the risk of infection. Rapid wound cleansing is essential for effective treatment. This can be achieved using surgical methods to remove necrotic tissue from the wound or conservative approaches using specialized dressings such as hydrogels, hydrocolloids or enzymatic cleansing techniques [20,25]. In addition, when deep burns exceed 30% of the body surface area, appropriate graft sources must be found. The gold standard for burn coverage is autologous split-thickness skin grafts (STSGs) from an uninjured donor site, which can be expanded with meshing or the Meek technology for complete wound coverage. Multiple skin harvesting sites for autografting are painful and require wound healing and additional treatment. However, when intact skin is inadequate, allografts and xenografts can provide only temporary wound coverage because they carry a risk of transplant rejection. Despite the many advantages of various skin grafts, current efforts are focused on developing specialized artificial skin substitutes using biological, synthetic, and biosynthetic materials to promote the primary and permanent closure of burn wounds, minimize scarring and reduce treatment time and costs [3]. 

### 4.1. Escharotomy

When circumferential eschar surrounds body structures, particularly the digits, extremities, abdomen, chest or neck, the underlying tissues are exposed to increased pressure [23]. This pressure is exacerbated by the development of edema during the first 4 to 6 h after injury [80]. As the interstitial pressure rises, it initially impedes the outflow of venous blood and subsequently impairs the inflow of arterial blood [79]. The result is dysfunction, ischemia or necrosis within or distal to the affected body structures, often with rapid onset. In the limbs, it can lead to the degeneration of nerves and muscles, resulting in long-term functional impairment or even the need for surgical interventions such as amputation. In the abdominal region, impaired blood supply to the bowel, kidneys and other internal organs results in the rapid onset of liver and kidney failure, bowel ischemia and reduced diaphragmatic mobility [79]. Abdominal compartment syndrome (ACS), caused by intra-abdominal hypertension (IAH), may also develop secondary to burn trauma. ACS can be treated via fluid resuscitation with continuous venovenous dialysis with ultrafiltration [81] or immediate surgical decompression via laparotomy. Escharotomy serves as a surgical procedure to relieve the constriction caused by the eschar, thereby restoring adequate perfusion and normal function to the affected tissues and organs. In most cases, a single incision does not provide the necessary relief from the constricting eschar. It is therefore common to make escharotomy incisions bilaterally on the trunk, or medially and laterally on each affected limb [82]. 

As part of the early management of burn wounds, the World Health Organization (WHO) recommends escharotomy in the first 48 h. Most clinicians believe that early surgical intervention (no later than 6 h after symptoms appear) is beneficial and that delaying the procedure leads to serious septic complications [82]. Only a few reports do not recommend early surgical treatment because of possible iatrogenic complications [83].

### 4.2. Debridement

The debridement process begins by cleaning the surrounding skin with soap, antiseptic or povidone iodine and shaving the hair if necessary. The wound is then washed and cleansed with 0.1% benzalkonium chloride or 0.05% chlorhexidine. Careful debridement techniques are used to minimize irritation. It is important to thoroughly remove detached non-viable epithelium from ruptured blisters and debris. In superficial partial-thickness burns, large blisters can be drained while small blisters can be left intact. For deep dermal burns, especially those caused by hot liquids, the loosely adherent epithelium should be removed [26].

Routine debridement has been shown to promote faster healing by reducing the activity of proteases that degrade growth factors. Wound cleansing within the first 24 h (<48 h) may reduce invasive burn infection, particularly in children [84,85]. Surgical debridement involves the removal of healthy tissue and the deformation of the wound contour, increasing the wound surface area. Moreover, it requires specialist care and intravenous analgesia [86]. Currently, other methods for removing dead epidermis are recommended, such as hydrosurgery, worm therapy, laser and special cauterization systems [87]. An interesting method for the non-surgical debridement of burns is using minimally invasive enzymatic debridement with proteolytic enzymes [88,89]. Topical debridement agents such as collagenase and papain are commonly used. Few enzyme preparations have been described in the literature, such as enzymes of bacterial (*C. histolyticum*, *B. subtilis*) and plant origin (papain, bromelain) [90]. The bromelain-based enzyme cleanser Nexobrid^®^, extracted from pineapple stems, is used for non-operative burn eschar removal [91]. The advantages and efficacy of this method are described in the papers by the Strużyna research group [86,92,93], which are based on the experience of a burn treatment center in Poland and their comparisons with the outcomes of patients who underwent surgical excision. The authors of the study point out the selectivity in identifying burn areas and the possibility of avoiding secondary operations and reducing the number of reconstructive operations due to scar contractures. According to Ziegler et al. [94], bromelain-based enzymatic debridement is preferred by almost 18% of specialized burn units. 

### 4.3. Topical Treatment

Studies have shown that the most common source of contamination in early burn wounds is the normal skin flora, particularly staphylococci, streptococci and methicillin-resistant *Staphylococcus aureus* (MRSA). *Pseudomonas aeruginosa* and *Escherichia coli* have been found in chronic wounds, usually in the deeper layers of the skin [95,96,97]. Studies show that chronic wounds can be colonized by many pathogens simultaneously, forming bacterial biofilms. Bacterial biofilms, surrounded by a protective extracellular polymer, maintain chronic inflammation, inhibit epithelial regeneration and protect bacteria from antibiotic therapy and the host’s immune response. 

Topical antimicrobials can be used in the form of creams, ointments and lotions. Burn centers usually have their own dressing preferences. The most commonly used topical antimicrobial agent is silver sulfadiazine (SSD), an effective antimicrobial agent against *Staphylococcus* and *Streptococcus* that has been widely used in burn wound management since 1968 [7,24]. A 2017 database analysis of randomized controlled trials (RCTs), which enrolled 5807 participants that included adult patients with second-degree burns occupying less than 40% of their TBSA, evaluated the efficacy of silver sulphadiazine (SSD) versus antiseptics: silver, honey, aloe vera, iodine, chlorhexidine or polyhexanide (biguanides), sodium hypochlorite, merbromine, etacridine lactate, cerium nitrate and *Arnebia euchroma* [98]. Statistical results provided low-certainty evidence that there may be little or no difference between the compared treatments. Previous studies by Fox et al. did not confirm the ability of SSD to inhibit bacterial growth, with particular emphasis on Gram-negative bacteria [99].

Mayer Tenenhaus et al. pointed out the possibility of pseudo scab formation at the wound periphery and impeded re-epithelialization under SSD [100]. The inhibitory effect of SSD on the development of granulation tissue after burns was also confirmed by a recent study from 2022. However, the authors of the study point out that SSD reduced the proliferation of bacterial colonies, both in the planktonic state and in the biofilm, and that the inhibition of tissue granulation was observed at the highest dose (800 µg/wound) [101].

Polyhexanide is used in many burn centers [94]. A 2017 study confirmed the effectiveness of polyhexanide-betaine gel compared to silver sulfadiazine in the treatment of partial-thickness burn wounds in terms of the healing time, infection rates, bacterial colonization rates and the pain score (*p* < 0.001) [102]. A recently published review article described the collected research on polyhexanide (poly(hexamethylene biguanide); PHMB) releasing membranes for wound healing [103]. In the 1980s, Johnson & Johnson developed cellulose-based non-woven membranes with PHMB for use as surgical drapes. Today, there are a number of commercially available PHMB-releasing dressings (PRWDs), for instance Fitostimoline^®^ Plus Gauze, Gemcore360°^TM^ PHMB Foam Border Dressing, Kerlix™ AMD, PuraPly^®^ AM and many others.

In addition to its antimicrobial effect, polyhexanide has other benefits, i.e., it reduces wound pain, reduces wound odor, increases granulation tissue formation and increases keratinocyte and fibroblast activity. Last updated in 2021, one of the best practice recommendation articles, which is a special publication of Wound Care in Canada, recommends the use of polyhexamethylene biguanide (PHMB)-containing ribbon gauze, gauze squares, transfer foam, gel and non-adherent synthetic contact layers, as one of the most common topical antimicrobials used in burn management [104].

Topical treatments include povidone-iodine [105,106], mafenide acetate/silver nitrate/sodium hypochlorite—with broad-spectrum activity against Gram-negative and Gram-positive bacteria—and nystatin, with antifungal activity. Bacitracin, neomycin, mupirocin and polymyxin B ointments are commonly used to treat superficial wounds. They may be used alone or in combination with petrolatum gauze to promote more rapid epithelialization. In addition, these ointments are often used in the routine care of superficial burns of the face. However, the aforementioned agents can also cause side effects such as leucopenia, delayed wound healing [23], hyperventilation, hyperchloremic metabolic acidosis [16] or auditory nerve toxicity [106], whereas the side effects of natural agents may be fewer and milder [107].

Aloe vera gel (*Asphodelaceae*) has a rich history of medicinal use dating back to ancient cultures. Numerous studies have been conducted over the years to investigate its pharmacological properties, including antibacterial, antiviral, anticancer, antioxidant and anti-inflammatory effects [107]. It is well known for its benefits to the skin, particularly in wound healing [108,109,110], and as an ingredient in cosmetic and pharmaceutical products. Clinical trials and reviews [109] have shown that aloe vera cream can accelerate the healing of second-degree burns, reducing wound size and healing time. It has also been found to reduce pain and be more cost-effective than 1% silver sulfadiazine cream [110]. 

*Albizia julibrissin* [111], *Arnebia euchroma* [112], *Betula pendula*, *Betula pubescens* [113], *Centella asiatica* [105], *Hippophaë rhamnoides* [114], and *Juglans regia* [115]—various studies have shown the advantages of these plants [22] over silver sulfadiazine cream, including faster healing times, less pain and burning, a greater percentage of wound epithelialization, and others.

## 5. Dressing

An appropriate dressing is essential for any wound and serves several purposes [26,78,116]. Firstly, it protects the damaged epithelium, minimizes bacterial and fungal colonization and helps maintain the desired position of the skin. Secondly, the dressing should be occlusive, to reduce heat loss and evaporation. Thirdly, it should provide comfort to the painful wound. In addition, it is important that the dressing has various properties such as the ability to control moisture, effectively remove exudate, facilitate gas exchange, have low skin adhesion, maintain mechanical stability, reduce wound necrosis, and be cost effective, non-toxic, biocompatible and biodegradable.

The choice of dressing depends on the characteristics of the wound. First-degree wounds with a minimal loss of barrier function usually do not require a dressing. Instead, topical balms, panthenol and aloe vera gels are used to relieve pain and maintain moisture. Cool showers and cold wet compresses can help relieve pain. Oral hydration is also important. Ibuprofen or paracetamol can be used for pain. Second-degree superficial wounds can be managed with daily dressing changes using topical antibiotics, cotton gauze and elastic bandages. Alternatively, temporary biological or synthetic dressings can be used to close the wound. Deep second-degree, third-degree and particularly severe fourth-degree burns require excision and grafting. The initial dressing should focus on controlling bacterial growth and providing closure until surgery is performed.

Wound dressings are usually made of natural polymers (chitosan, cellulose, fibrin, elastin, hyaluronic acid, dextran, elastin, alginate, collagen and gelatin) and/or synthetic polymers (PVP, PEO/PEG, PHEMA, PVA, PU, iPGA, PLGA and PLA) [117] in various forms such as hydrogels, films, nanofibers, foams, topical preparations, wafers, transdermal patches, sponges and dressings [118,119,120,121,122,123,124]. Many dressings impregnated with silver and other antimicrobial agents are commercially available [116,125]. Aramwit et al. [126] compared in vitro the efficacy of some of them, namely, Urgotul SSD(^®^), Bactigras(^®^), Acticoat(^®^), Askina Calgitrol Ag(^®^) and Aquacel Ag(^®^) in terms of their antibacterial activity and ability to maintain the appropriate level of moisture. In vitro analysis was carried out against methicillin-sensitive and -resistant *Staphylococcus aureus*, *Bacillus subtilis*, *Escherichia coli* and *Pseudomonas aeruginosa* using Mepitel(^®^) as a control. It was found that although they were all bactericidal, they differed along that spectrum and in speed of action. Acticoat(^®^) showed the broadest spectrum of activity, while Askina Calgitrol Ag(^®^) showed the broadest spectrum of activity due to the absorption and release of moisture.

In the case of partial-thickness superficial burns, when the epidermis and dermis are damaged, after cleaning the wound, many centers use biosynthetic epithelial substitutes, e.g., Biobrane produced by Dow Hickam/Bertek Pharmaceuticals Inc., Sugarland, TX, USA, or AWBAT-S (Advanced Wound Bioengineered Alternative Tissue-Superficial), manufactured by Aubrey Inc., Carlsbad, CA, USA. Both substitutes are made of silicone and nylon foil and collagen peptides, but they differ in the arrangement of pores in the silicone membrane. AWBAT^®^ became commercially available after its FDA approval in 2009. Biobrane was developed in 1980 by Tavis. Unfortunately, despite numerous articles on the subject and many years of positive experience regarding the usefulness of these synthetic, biocomposite membrane dressings in the treatment of intermediate thickness burns, and sometimes of full-thickness excisions, they are not available in many parts of the world [127]. Greenwood compared both dressings in a randomized study [128]. Table 2 shows examples of the different categories of commercially available dressings.

The type of dressing influences burn wound healing. Wasiak et al. [129] evaluated the effects of different dressings (hydrocolloid; polyurethane film; hydrogel; silicone-coated nylon; biosynthetic skin substitute; antimicrobial; fiber; and wound dressing pads) on superficial and partial-thickness burns. The authors searched several databases, including randomized controlled trials (RCTs). Studies that pooled the results of different interventions showed the advantage of biosynthetic dressings.

Hydrocolloids are substances that have the ability to form gels or thick, viscous dispersions when in contact with water [130]. They have the ability to absorb small to moderate amounts of exudate, retain moisture in the wound, create a mildly acidic, low-oxygen environment, facilitate capillary growth and accelerate the wound healing process [131]. Some studies suggest the superiority of hydrocolloid dressings over paraffin-based gauze in the treatment of mixed dermal burns in pediatric patients. This is evidenced by lower debridement and grafting rates, a smaller graft surface area and shorter hospital stay [132].

Hydrogels are modern dressings made from natural or synthetic polymers that form highly water-absorbent networks [133,134]. They have unique properties that make them ideal for patient recovery, such as sensitivity to the body’s environment, flexibility and a high moisture content [135]. These dressings have multiple functions, including cooling, wound coverage and heat dissipation through convection and evaporation [136]. Some hydrogel dressings even contain anesthetic, nutritional or anti-inflammatory agents [134].

Petrolatum gauze—a very basic dressing commonly used for large superficial burns [137]. Antiseptics such as chlorhexidine may be added. If the gauze does not stick, this indicates a deeper burn that may require treatment with topical antibiotics or surgery.

Honey-based dressings—honey, a thick syrup rich in carbohydrates, has been used in traditional medicine since ancient times [138,139,140]. It is now widely recognized for its antibacterial, antiparasitic and analgesic properties, particularly in the treatment of respiratory infections. When used in the treatment of burns, honey offers several advantages, including creating a moist environment, maintaining the integrity of the burn surface by not adhering directly to it, and acting as a bacterial barrier to prevent cross-contamination [24,139]. A study in mice by Febriyenti et al. demonstrated the efficacy of honey in burn healing [139]. Given the potential benefits of natural ingredients in modern medicine, the inclusion of honey in burn treatment is desirable due to many of its properties.

### 5.1. Dressings Made of Natural Polymers

A comprehensive review on chitosan and cellulose hydrogels, and their derivatives, for wound healing has recently been published [141]. Biomaterials based on chitosan, a deacetylated derivative of chitin, are widely used in the treatment of wounds [142]. Chitosan is a basic polysaccharide with antifungal, antimicrobial and antioxidant properties. It can be in the form of microparticles [143]. Chitin and its derivative, chitosan, are widely available and inexpensive biological materials derived from fungal cell walls, insect exoskeletons and invertebrate skeletons [144]. Chitin is a biocompatible, biodegradable, non-toxic, anti-bacterial, non-antigenic and humectant material [145,146]. Chitosan plays a crucial role in wound healing by promoting homeostasis through its ability to bind to red blood cells, facilitating rapid blood clotting [147]. It also increases fibroblast proliferation, modulates inflammatory cell functions, promotes granulation and facilitates cell organization [135]. When used as a semi-permeable biologic dressing, chitosan maintains a moist environment, optimizes healing conditions and prevents scarring and wound contamination. The scientific literature over the past 40 years has confirmed the efficacy of chitin and chitosan as biomaterials, supported by clinical and veterinary studies, resulting in significant reductions in treatment time and minimal scarring in various animals [146]. Recently, in [148], a temperature-sensitive chitosan hydrogel (TCTS) was synthesized. In the discussion of the mechanism of action of the TCTS, the arose the possibility of (i) the interactions of chitosan molecules, positively charged due to the presence of protonated amine group NH_3_^+^, with negatively charged microbial cell membranes providing an increase in the permeability of the bacterial membrane, (ii) binding to bacterial DNA, (iii) the binding of essential metals required for bacterial metabolism (Ca^2+^, Mg^2+^), and (iv) the formation of barriers on the surface of bacterial cells, blocking the entry of nutrients and oxygen. The chitosan-based hydrogels can also be used as scaffolds for loading antimicrobial drugs such as antibiotics and silver sulfadiazine [149].

Silk fibers from the silkworm Bombyx mori have been used as surgical sutures and in the manufacture of dressings for almost 30 years [150,151,152,153,154,155,156,157,158]. Fibroin proteins, which are biocompatible and biodegradable, have been used in tissue engineering, in contrast to another silk fiber protein, sericin, which some authors suggest may cause increased inflammation, allergic and immunogenic reactions [153,154,155]. In vivo studies in animal models such as Sprague Dawley mice and rats and Wistar albino rats have demonstrated the efficacy of silk fibroin (SF) dressings in the treatment of second- and third-degree burns [156,157,158]. SF dressings were found to be more effective than the commercially available polyurethane hydrocellular dressing foam Medifoam^®^ (Genewel Co., Ltd., Gyeonggi-do, Korea), Purilon gel (Coloplast A/S, Inc., Humlebaek, Denmark) or ordinary medical gauze. The authors of the study highlight the faster healing of wounds treated with SF hydrogel, their reduced inflammation, greater vascular density and M2 macrophage infiltration.

### 5.2. Dressings Made of Inorganic Materials

Non-biological dressings are a safe option for closing superficial burns. They create a moist environment that promotes faster epithelialization and require fewer dressing changes, reducing pain and anxiety for patients. Non-biological products are also more cost-effective than biologically engineered skin substitutes.

Inorganic materials, including silicate minerals (mineral clays, kaolin, zeolites), silica-based materials (mesoporous silica, mesoporous bioactive glasses, diatomaceous silica and their composites), metal-containing materials (Ag, Cu, ZnO NPs), phosphates (hydroxyapatite, tricalcium phosphate) and carbon derivatives (carbon nanotubes, graphene oxide) are used in medical applications for their hemostatic properties [159]. 

Microporous materials, i.e., zeolites, can be used as sustained release drug delivery systems. An example of the use of zeolites is in chitosan/zeolite composite films loaded with silver sulfadiazine [160], which were effective against Candida albicans and some Gram-negative bacteria.

### 5.3. Dressings Made of Nanomaterials (NMs)

NMs have antibacterial properties, i.e., nanoparticles of precious metals, gold, silver and others, are only vectors delivering therapeutic agents. Nanoparticles offer a wide range of properties that can be designed by adjusting the type of material, its size, the functional groups on the surface, surface charge (zeta potential) and polydispersion [161]. There are various forms of nanomaterials, including organic NMs such as nanopolymers, nanocapsules, nanoemulsions, nanogels, micelles, liposomes, nanocolloids, solid lipid-based NPs, and inorganic ones such as nanocarbons, metal and metal oxide NPs (Au, Cu, ZnO, Ag, TiO_2_, magnetic NPs and quantum dots). In recent years, other forms of nanomaterial dressings have been introduced, such as nanofibers (NFs), nanosheets and 3D scaffolds [162,163,164]. Although metallic nanomaterials exhibit efficient antibacterial activity, their potential toxicity should be taken into account when using them for in vivo applications. Although AgNPs are known to treat burn wounds, the potential release of Ag^+^ ions from the nanomaterial, which are toxic to mammalian cells, may cause argyria and argyrosis in humans, which should be considered. Some metal/metal oxide nanoparticles such as AgNPs and ZnONPs may enter the bloodstream and cause hemolysis. Polysaccharides or other biologically active substances can be placed on the surface to prevent the release of NPs and contact with blood cells. The solution to this problem is to cover the Ag/AgCl NPs with graphene. This solution has been tested in animal models [165]. Jiji et al. [166] anchored silver nanoparticles in bacterial cellulose (BC-PDAg); the obtained BC-PDAg exhibited antibacterial activity and promoted the processes of wound healing thought to accelerate fibroblast proliferation, granulation tissue formation, angiogenesis and re-epithelialization. In contrast, polymeric nanomaterials such as chitosan have good biocompatibility in addition to bactericidal properties [167]. Recently, multimetallic nanomaterials have been used in the treatment of wounds. An example is the work of Li et al. [168], which describes the use of bimetallic CuCo_2_S_4_ NP to effectively destroy MRSA biofilms in vitro and accelerate burn healing. The activity of CuCo_2_S_4_ NP was compared with that of peroxidase. The effective antibacterial activity of metal/metal oxide NPs is ensured via photocatalysis. Under the influence of UV radiation, free oxygen and hydroxyl radicals are formed on the surface of NPs, which kill microorganisms. Wang et al. [169] prepared multimetallic NPs consisting of CuO and ZnO and Au, which, when exposed to laser light at a wavelength of 635 nm, killed almost 100% of *S. aureus* and *E. coli* (97.5%) after 10 min of exposure. Ye et al. reported the antibacterial efficacy of CuO@AgO/ZnO NPs exposed to near infrared (NIR) light against *S. aureus* and *Pseudomonas aeruginosa* [170]. In addition to the nanopreparations already used in clinical practice, new studies are being published. A comprehensive review of nanoproducts for topical application in the treatment of skin disorders has recently been published by Raszewska-Famielec and Flieger [171] and others [172,173,174,175,176]. An example of an innovative treatment for burn wounds is the therapy proposed by Li et al. [177] using mesenchymal stem cells (MSCs) labelled with Fe_3_O_4_@polydopamine (Fe_3_O_4_@PDA) NPs. Cell and rat studies confirmed the increase in the migratory capacity of MSCs after intravenous administration. 

Electrospinning is a technique for producing fibrous structures for tissue engineering and wound dressings. Synthetic polymers, i.e., polyvinyl alcohol (PVA) and chitosan (CS), are most commonly used to produce tissues that provide high mechanical stability. Biological properties are achieved by incorporating bioactive agents, such as usnic acid, which is an alternative to antibiotics in the treatment of burns. Stoica et al. [178] described the fabrication of a nanofibrous, electrospun mesh based on PVA, chitosan and usnic acid with applications in wound healing. In a study in 2023 [179], bioactive nanofibrous zinc silicate Zn_2_SiO_4_ (ZS) nanoparticles with spindle-like morphology were synthesized via a hydrothermal method. The in vivo results in an animal model confirmed the efficacy of the ZS nanoparticle scaffold for re-epithelialization and the reconstruction of cutaneous neurovascular networks.

The combination of nanotechnology with herbal remedies is a relatively new approach to burn treatment [180]. Nanocarriers enhance the bioavailability of herbal constituents and ensure their sustained release. Phytochemical-based nanopreparations developed for the treatment of burns are summarized in Table 3.

In Saito et al.’s study [198], polymeric nanosheets prepared with a poly(vinyl acetate) loaded with the antibiotic tetracycline were investigated as antimicrobial agents. The authors confirmed the efficacy of TC nanosheets in the treatment of full-thickness burn wounds infected with *P. aeruginosa* in an animal model. Tetracycline, one of the most effective antibiotics for the treatment of burn wound infections, was used to enrich a citric acid functionalized chitosan hydrogel [199]. Burn dressing designs with commercial potential are still being published. An example is research using another antibiotic, minocycline, in dressings based on a nanocomposite film of polyvinyl alcohol (PVA) and halloysite nanotubes (HNT) [200]: a composite membrane based on halloysite nanotubes/poly(lactic-co-glycolic acid)/chitosan [201] and hydrogel with sodium alginate (PVA-SA) [202]. Gentamicin has been used in the manufacture of other burn dressings, e.g., a nanobiocomposite membrane containing chitosan [203], gelatin microspheres embedded on silk fibroin [204] and porous poly(DL-lactic-co-glycolic acid) embedded on a layer of spongy collagen [205]. Gentamicin has also been tested as an adjunct to emulsions such as BSA, SPAN and BSA2, which have different release kinetics [205] and were effective in treating infection and promoting healing at the stage of angiogenesis, epithelialization and collagen formation. Other antibiotics used in wound dressings include datoromycin and fusidic acid, which have been shown to be effective as a component of hybrid lipid–polymer NPs (LPHNs) in the treatment of MRSA infection [206].

Due to antibiotic resistance, and an estimated 95% of Pseudomonas are antibiotic resistant [207], synthetic and natural antimicrobial peptides (AMPs) are an attractive alternative for the treatment of burn wound infections. AMPs such as PXL150 [208], IRIKIRIK, IK8L [209] and WRL3 [210] have been tested in the treatment of infections due to their bactericidal activity against MDRP, *P. aeruginosa, S. aureus* and their anti-inflammatory and healing properties. Although it is possible to embed PXL150 [208] in hydroxypropylcellulose gel and L12 [211] can form a hydrogel network with DNA nanostructures, the binding of AMPs to polymeric scaffolds requires further research.

The treatment of burn wounds involves not only the fight against bacterial infection, but also the fight against the inflammatory response and the low expression of various growth factors (GFs). A variety of GFs have been used to treat burn wounds, including keratinocyte-forming GFs, transforming growth factor beta (TGF-β), epidermal growth factor (EGF), nerve growth factor (NGF), basic fibroblast growth factor (bFGF), vascular endothelial growth factor (VEGF) and platelet-derived growth factor (PDGF) [18,212]. Typically, GFs are applied to the surface of the burn wound to promote re-epithelialization, neovascularization and granulation tissue formation, thereby reducing healing time. The problem remains the poor stability of GFs, which is solved by entrapping GFs in nanomaterials [213,214]. Nanotherapeutics used in the treatment of burn wounds show very good antimicrobial activities, even against resistant strains of microorganisms, and are capable of accelerating the regeneration of burned skin, which has been demonstrated in both in vitro and in vivo models using human tissue and animals such as mice, rats, rabbits, dogs and piglets [95,213,215,216].

## 6. Excision

It was not until the 1970s that early excision and grafting was introduced as the standard of care for burn injuries [217]. Early burn excision has clear benefits, including increased survival, reduced infection rates and shorter hospital stays. The early removal of burned tissue also appears to reduce the risk of hypertrophic scarring [18,26].

Surgeries can be spaced 2 to 3 days apart until all burned tissue is removed and the wound is covered. Temporary coverage with biologic dressings or cadaver grafts is possible until autogenous donor sites are available.

There are two techniques: tangential excision and fascial excision. Tangential excision involves the sequential removal of layers of burnt and necrotic tissue until viable tissue is reached. It can result in blood loss and difficulty in assessing wound viability when large areas are removed. Fascial excision removes burnt and subcutaneous tissue down to the muscle fascia, removing subcutaneous fat, blood vessels and nerves and providing better hemostasis. In some cases, veins may be preserved to maintain venous return. For deep burns involving muscle, excision should reach healthy tissue with active bleeding and muscle contraction. However, it may inadvertently remove viable tissue and cause contour deformity and lymphoedema in the excised areas.

It can be noted that the majority of previous observational and randomized studies of excision and skin grafting are those performed within 24–72 h after injury [218,219,220]. Most specialized burn centers consider early excision of the wound within the first 48 h post-burn, and the use of a graft, to be beneficial [82]. 

Wu et al. [221] conducted research on 157 children admitted to hospital between 1995 and 1999 due to burns covering 40% of their body surface and more than 10% of which were full-thickness burns. Three populations of patients who underwent surgery on days 0–2 (n = 86), 3–6 (n = 42) and 7–14 (n = 29) after burn injury were studied. Study results showed that delayed surgical intervention resulted in greater wound contamination, invasive infections (*p* < 0.001), sepsis (*p* = 0.04) and was associated with longer hospital stays (*p* = 0.008). A recently published retrospective study using a Japanese patient database showed that excision or skin grafting within 2 days of burn injury was not associated with increased in-hospital mortality compared with subsequent surgery in patients with severe burns [222]. The study included a population of 2362 divided into groups of patients who underwent excision or skin grafting within 2 days of admission, and a second group who underwent surgery within 3–7 days of admission. There was no statistically significant difference in mortality between the early surgery group (15.9%) and the delayed surgery group (17.2%; *p* = 0.70).

## 7. Permanent Wound Coverage

Significant progress has been made in reducing mortality rates through the implementation of increasingly aggressive early tangential excision of burn tissue and the prioritization of early wound closure, primarily with skin grafts [7]. 

Skin substitutes are a group of materials designed to close the wound temporarily or permanently, to protect it mechanically and against bacterial colonization, and to take over its function. Such a material should be free of allergens, sterile, and provide adequate flexibility, durability, and the ability to integrate with the patient’s tissue. In addition to synthetic materials, it is possible to construct biomaterials. Skin substitutes can be temporary dressings to speed up the healing of burns, or advanced tissue-engineered products that heal the injury permanently. Skin substitutes typically contain a carrier/matrix through which nutrients, growth factors and skin cells, i.e., epithelial cells, fibroblasts, melanocytes, etc., are delivered. The purpose of such a dressing is to integrate with the substrate and create new autologous skin.

In addition to composite skin substitutes, specific devices have been developed, such as the ReCell for spraying a suspension of skin-resident cells, the RenovaCare gun for delivering skin cells or stem cells, and a 3D printer that prints a matrix with the structure of the skin and epidermis from the supplied autologous cells [223].

One of the best-known classifications of skin substitutes is the Davison–Kotler classification [224]. The criteria included in this system are the composition of the skin substitute, the structure, the type of biomaterial and the duration of wound coverage. Examples of biomaterials available on the medical market are shown in Table 4.

### 7.1. Autografts

Autogenous grafting requires a fresh and uncontaminated wound bed, otherwise the wound must be temporarily closed, e.g., with an allograft. A split-thickness skin autograft is recommended for functional areas if available and the patient’s condition permits. A meshed skin autograft can be used for relatively extensive burns where graft availability is limited, but may result in patchiness and poorer functional outcomes [26,106]. A skin autograft is certainly the gold standard for replacing damaged skin, but it is not possible in patients with a significant percentage of their TBSA damaged. The order of excision, or the decision on which areas should be prioritized and which areas are useful as graft donor sites, is up to the surgeon. The primary goal is patient safety. Typically, areas such as the front or rear torso or limbs will be excised first. The size of the resection depends on the size of the available autograft [230]. Usually, no more than 40% of the TBSA can be excised at one time [231]. When the TBSA burned is large, sheet grafts are used on more visible parts of the body such as the face, neck or hands, to reduce the visibility of scarring. Large burn wounds are usually covered with mesh skin grafts because the removed skin can be stretched to cover a larger area. Engrafting is the process of revascularization, where the graft receives nutrients via diffusion from the wound bed as it has no vascular connection to the tissue. Revascularization typically begins about 48 h after graft placement and involves neovascularization and inosculation, the direct connection of recipient vessels to the graft. Concurrent with revascularization is the organization phase, during which the graft integrates with the wound bed [26].

Over the course of five days, the skin graft adheres via imbibition, inosculation, and capillary in-growth [16].

Skin grafts are categorized according to thickness: split thickness (partial) or full thickness. Split-thickness grafts can be thin, medium, or thick, affecting contraction at the recipient site and dermal deficit at the donor site. Thicker grafts contract less at the recipient site but have a longer healing time and increased risk of hypertrophy [26].

#### 7.1.1. Split-Thickness Skin Graft (STSG)

A STSG involves the epidermis and the partial thickness of the dermis. It is widely recognized as the gold standard for achieving a durable and permanent closure [79,232]. Secondary contraction is greater than primary contraction. The donor site typically heals within a range of 10 days to 2 weeks, which may vary depending on the thickness of the graft [16].

Grafts can be meshed or unmeshed (sheet grafts), with sheet grafts being preferred for aesthetic reasons, particularly in exposed areas such as the face, hands and forearms. Meshing is necessary in larger burns to cover all affected areas and can be performed in various ratios (even 6:1), although 2:1 is common. Meshing allows fluid drainage, minimizing complications such as seroma and hematoma, and provides expansion for greater wound coverage.

Various techniques can be used to secure skin grafts to the wound bed. Staples are commonly used for large areas, while sutures are advantageous in children, as absorbable sutures eliminate the need for removal [26].

#### 7.1.2. Full-Thickness Skin Graft (FTSG)

A FTSG involves the epidermis and all of the dermis. Due to the presence of elastic fibers, the primary contraction is greater than the secondary contraction, which is why it is often used to cover wounds on the face and hands. The result is also more cosmetically pleasing. The donor site is primarily closed [16]. Common donor sites include the axillary, supraclavicular, periauricular and inguinal [16]. A team from a burn center in Krasnodar, Russia, presented their long-term experience in evaluating the efficacy of full-thickness skin grafts for facial burns [233]. A total of 97 patients with facial burns were divided into a group treated with full-thickness skin grafts (n = 42) and a group treated with split-thickness skin grafts (n = 55). A positive cosmetic effect and no indications for reconstructive surgery were achieved with the full-thickness skin graft approach. However, other options have been proposed for facial scars, such as a thin partial-thickness skin graft plus a dermal substitute (Integra or Matriderm), a pre-expanded flap from the surrounding area (shoulder, back), or an allograft requiring further immunosuppression [234].

#### 7.1.3. Cultured Epithelial Autograft (CEA)

CEA involves culturing a sample of the patient’s skin in the laboratory to produce epithelial cells, which are then applied to cover a fragile wound. The use of these techniques has the potential to reduce the amount of donor skin needed to treat large burns, resulting in a significant reduction in healing time for both the donor site and the burn area. In addition, these techniques may increase overall graft success and improve scar quality [79,232]. However, they have certain limitations, including increased susceptibility to infection and the absence of a dermal layer, resulting in fragile healed skin and severe scarring [79,232]. Unlike split-thickness skin grafts, a CEA lacks dermal matrix tissue. When applied to full-thickness burns without dermis, the healed wounds become stiff and lack elasticity. A CEA placed directly on the muscle or subcutaneous tissue gradually forms a basement membrane, sometimes taking up to six months. The absence of a well-formed basement membrane makes the epidermis susceptible to blistering and shearing. The reopening of healed wounds is common after CEA application. The growth of CEAs is slow and costly, requiring a biopsy sample from the patient and taking several weeks to develop. Carsin et al. [235] showed that cultivated epithelial autografts were useful for extensive burn coverage in severely traumatized patients. This conclusion was based on a five-year single-center study with 30 patients. Some centers have experimented with cultured epidermal allografts for the treatment of burn wounds, with varying degrees of success [19], but the use of these techniques is mostly limited to large burn patients who have no other therapeutic options [79,232].

### 7.2. Flaps

Dermal substitutes offer improved functional outcomes when integrated into the wound, although engraftment rates have been problematic. By replacing lost dermis in deep second- or third-degree burns during initial treatment, these materials may reduce the need for future reconstructive surgery. They also have the potential to prevent the extensive scarring and poor skin elasticity often seen when thin skin grafts are used to cover burns. Despite the technical challenges associated with these materials, their ability to prevent the functional and cosmetic deformity caused by scar contracture justifies their use in many clinical scenarios [19].

For deep burns, where deep anatomical structures such as blood vessels and nerves are exposed, a local pedicle flap is used to protect the tissue and promote healing. An island flap may be used for specific purposes. Microsurgical techniques have enabled the use of tissue and organ transplantation in surgical treatment. Free skin flaps, musculocutaneous flaps and composite tissue flaps have become popular in plastic surgery and are now used in severe burn cases where the preservation of deep anatomical structures is critical. A free skin flap involves anastomosing the blood vessels of the flap to those of the recipient site. Composite free flaps are most effective in the early postoperative period. The results of these procedures vary depending on the stage of burn recovery [79,106].

## 8. Temporary Wound Coverage

Biological dressings have multiple functions, such as protecting wounds from contamination, reducing pain and minimizing fluid loss. They can also potentially deliver growth factors to promote wound healing [24].

### 8.1. Allografts and Xenografts

Biological skin substitutes include materials derived from individuals of a different species (xenografts) and those of the same species (allografts). The latter can be obtained from living donors or from cadavers [236]. For patients with a significant percentage of their TBSA damaged, a skin autograft is not possible. In this case, the solution is to temporarily cover the wounds with skin allografts [237,238]. It is believed that allogeneic skin is an excellent but temporary biologic dressing that is discarded within 1–2 weeks or has to be removed because of infection. However, there are occasional reports of studies demonstrating that human leukocyte antigen (HLA)-matched allografts do not need to be rejected and may even survive permanently [239]. Skin banks, where skin can be stored in frozen liquid nitrogen for up to five years, have been established in many countries to provide a supply of skin allografts [240]. Recently, a team from the Department of Plastic, Reconstructive and Aesthetic Surgery at the Medical University of Vienna described their experience with the skin bank program [237].

Traditional biological products such as xenografts and allografts are commonly used in burn care [24]. Fresh or frozen cadaveric split-thickness skin is widely used as a biologic dressing. It remains viable when properly handled and promotes revascularization when applied to a healthy wound bed. However, it is subject to rejection after 1–2 weeks. Porcine skin and freeze-dried cadaver skin have different properties and may not revascularize or provide the same benefits. Allografts have shown promising results in trials, even outperforming silver sulfadiazine in healing partial-thickness burns. Xenografts can adhere to the surface of partial-thickness burns and aid in eschar debridement [24]. They also reduce pain in burn patients [241,242].

However, in both cases, there is a risk of disease transmission, immunological reactions [242,243], high cost and the need for cryopreservation of the grafts [19].

Despite this debate, Choi et al. [244] demonstrated the significant statistical utility of cadaveric skin allografts in patients with burns involving >30% of their TBSA. The study involved 1282 patients hospitalized between 2008 and 2016 in Korea. Statistical analysis showed a significant difference in mortality between two groups, one of which (n = 698) underwent cadaveric skin allograft, while the other (n = 584) received conventional treatment. The 90-day mortality rate was 35.3%. The mortality rate in the study groups was 453 and 1282, respectively.

### 8.2. Human Amnion

Placenta can be used as a temporary dressing for partial-thickness and excised burns when an autograft is not available, particularly in resource-limited settings [24]. It acts as a biological barrier, reducing pain and providing transparency. It is also easy to apply and remove. The collection, preparation and storage of placenta can be challenging. The dressing requires frequent changes every two days and is prone to disintegration. There is also a potential risk of disease transmission and it is a costly option [19]. Another treatment option is to use the amniotic membrane, which is on the inside of the placenta. During pregnancy, the amniotic membrane secretes various epithelial renewal, anti-angiogenic and anti-cancer factors, as well as immunomodulatory and anti-inflammatory factors. Amniotic membrane has antiviral and bacteriostatic properties. In the 20th century, the autotransplantation of amniotic/chorionic tissue began to be used in the treatment of various wounds [245].

### 8.3. Artificial Skin (Biosynthetic Dressings)

Biosynthetic dressings have gained popularity due to their ability to accelerate wound healing, reduce pain, minimize scarring and provide bioactive dermal-like properties [19]. They are typically used until the wound is completely healed, usually within 10 to 14 days. However, the improper use of biosynthetic dressings in deep partial-thickness burns can lead to higher infection rates. They show positive results in the treatment of superficial partial-thickness burns, but care must be taken to prevent infection in deeper burns. They also demonstrate faster healing and less pain in mid-dermal burns after debridement, as observed in prospective trials [24].

### 8.4. Fish Skin Grafts (FSG)

The demand for cellular and tissue-based products for severe burn wounds is in-creasing, especially in conditions with limited donor sites for the autogenous transplant of tissue from other areas of intact skin. In a study by Stone R. et al., the authors described that acellular fish skin grafts (FSG) outperformed fetal bovine dermis in terms of wound healing [246]. Fish skin showed superior results with faster integration and re-epithelialization, without increased contraction. In a study by Jaechul Yoon et al. [247], a comparison of FSG (Kerecis^®^) with a bovine collagen skin graft (ProHeal^®^) was performed in 52 patients with acute burns. It was found that healing was faster in the Kerecis^®^ treatment group than in the other treatment group. A product called Kerecis^TM^ Omega3 Burn was used successfully in the treatment of 10 patients who received split-thickness skin grafts as part of a pilot study at the Burn Center at the Queen Elizabeth Hospital in Birmingham [248]. Current evidence proves that this approach leads to accelerated wound healing, reduced pain, a decreased need for frequent dressing changes and improved long-term outcomes [243,249]. Fish skin is a valuable collagen source, especially type I78 and III, fibrin, proteoglycans and glycosaminoglycans, omega-3 polyunsaturated fatty acids, eicosapentaenoic acid and docosahexaenoic acid [243,250,251]. Furthermore, contrary to the mammalian allo-/xenografts, it does not carry the risk of disease transfer such as bovine spongiform encephalopathy and variant Creutzfeldt–Jakob disease [243,246,249]. 

FSGs are derived from species such as Nile tilapia (*Oreochromis niloticus*) or the wild North Atlantic cod (*Gadus morhua*). The FSG dressings were approved by the US Food and Drug Administration (FDA) in 2013. The Kerecis^®^ Omega3 dressing is the only certified FSG currently commercially available and is manufactured by Kerecis, Isafjordur, Iceland [249,250]. The long-term effects of treating burn wounds with FSGs were investigated by Wallner et al. [252]. A retrospective case–control study was conducted on 12 patients with superficial or deep burn wounds. The wounds were debrided enzymatically (NexoBrid™) followed by a FSG, Suprathel^®^ or STSG. The results were collected for 1 year after the burn injury. The results of the healing rate, water retention, elasticity, skin thickness and pigmentation, pain relief and itching were found to be better with fish skin compared to treatment with STSG or Suprathel. The authors’ findings support the possibility of the use of fish skin as a promising therapeutic option for enhanced wound closure in deep-partial thickness burn wounds after non-surgical debridement. 

Advances in biotechnology and regenerative medicine have enabled the development of Kerecis FGS products, which are available in many shapes, sizes and variants (standard, micro, nano, 2:1 mesh). In the future, the possibility of using other fish species to produce transplants should be considered. In the case of FSGs, the possibility of an allergy or hypersensitivity to fish material in some patients remains a problem.

Acellular fish skin shows promise as a cost-effective alternative for the treatment of superficial and deep partial-thickness burns. However, further research with large cohorts is essential to fully understand the potential and benefits, as well as the unknown limitations, of this promising approach [243].

## 9. Additional Actions

### 9.1. Maggot Debridement Therapy (MDT)

Larval debridement therapy, in addition to surgical excision, effectively removes necrotic tissue from wounds, dissolves fibrin clots and degrades laminin, type I and type III collagen and fibronectin [253], but also has bactericidal and bacteriostatic effects and promotes the healing process. This method is safe, effective, user-friendly and cost-effective, but requires expertise from healthcare professionals and acceptance from patients and carers [254]. In 2018–2023, 126 articles were published in the PubMed database on *L. sericata* larvae therapy [255,256,257,258,259,260,261,262,263], of which 6 were review articles [264,265,266,267,268,269]. The results show that 70–90% of the wound surface can be successfully cleansed, with minor bleeding and epidermal irritation reported as the only complications, while wound granulation increased. The use of *Lucila sericata* larvae (*Phaenicia sericata*) for debridement reduces the time taken to remove necrotic tissue and promotes faster granulation. The enzymes arginase, leucine aminopeptidase, collagenase and proteases (serine proteases and metalloproteinases) are responsible for the wound cleansing process. Recently, amino acids such as L-histidine, 3-guanidinopropionic acid and L-valinol have been identified, in larval secretions, to promote the proliferation of human endothelial cells [270]. After proteolytic digestion, the phagocytic activity of leukocytes and increased oxygen tension are observed [271]. The vibrating movement of the larvae on the wound surface promotes neoangiogenesis and granulation. The antibacterial compounds phenylacetic acid and phenylacetaldehyde are produced in the larval gut by symbiotic microbes (*Proteus mirabilis*) [272].

Larvae are also able to produce the bactericidal lucifensin, lucifensin II, lucilin and alpha-methoxyphenyl (MAMP) [273], and ammonia, calcium carbonate and ammonium carbonate, which alkalize the wound environment. Larval secretions interfere with the biofilm formation and degradation of *Staphylococcus aureus*. Biofilm disruption occurs at a concentration of 20 µg [274]. Meta-analyses based on observations from many countries, including China, the UK, the USA, Israel, Malaysia and Thailand, have shown that wound debridement with MDT is faster than with conventional treatments [275]. The barrier to the widespread use of this method is undoubtedly the use of worms, so there is a current trend for using transgenic larvae to produce various molecules that accelerate healing. One example is the production of PDGF-BB protein in maggot excretions/secretions after induction, developed by Linger et al. [276].

Worm therapy is mostly used for chronic wounds. The beneficial effects of Coriaria Sinica Maxim’s and *Lucilia sericata* maggots extracts on burn wounds have been tested on animal models in rats with deep second-degree burns [277,278]. 

A case report of the treatment of a 29-year-old man who suffered from extensive fourth-degree electrical burns using MDT was described in 2017 [279]. MDT successfully prepared the wound bed for skin grafting, without any interactions, allergies or inflammation.

The first clinical trial performed to evaluate the effectiveness of MDT in the treatment of full-thickness burns was reported in 2023 [280]. The study included 31 patients with full-thickness burns (degree III). In total, 15 cases qualified for larval treatment, while 16 cases received conventional treatment including acute wound debridement, silver sulfadiazine, and antibiotic therapy. The authors of the study demonstrated the benefits of using MDT, which ensured a multiplicity of changes; reduced necrosis (2nd day *p*  = 0.028, 4th day *p*  = 0.023), favorable differences in granulation (*p* < 0.001) and a shortened healing time (*p*  < 0.001) in comparison with the control. The benefits of MDT were particularly evident in those with highly necrotic burns (necrosis > 50%) (*p*  < 0.001).

The efficacy of MDT on burn wounds, as an alternative treatment, has been reviewed by Barbara Irish from Pacific University, using MEDLINE-Ovid, Web of Science, CINAHL and Google Scholar, as her thesis dissertation [281].

### 9.2. Negative Pressure Wound Therapy (NPWT)

The use of negative pressure wound therapy, also known as Vacuum-Assisted Closure (V.A.C.), has shown its potential to halt the progression of partial-thickness burns. It is a mechanical method that uses sub-atmospheric pressure to treat wounds [282,283]. The application of low pressure is tailored to the specific wound type, surface and factors that may affect the complexity of treatment, such as wound infection [284]. During therapy, cells undergo mechanical stretching, which stimulates cell proliferation and accelerates wound healing [283,284,285,286,287]. The efficacy of this method is due to its multifaceted approach, including increased local blood flow [287], enhanced collagen synthesis and mechanisms that promote angiogenesis, resulting in improved granulation and epithelialization, thereby facilitating the healing process, promoting graft acceptance and reducing the risk of multiple skin grafting procedures [287]. In addition, the beneficial effects of the therapy are further enhanced by factors such as reduced local swelling and the reduced presence of harmful microorganisms in the wound [25,282].

Although it is not currently a widely used treatment, it is possible that negative pressure wound therapy may become more widely used in the future.

The therapy has been validated for the treatment of pseudoepithelial hyperplasia (PEH) as a secondary reaction to burns [288]. Three cases of patients with second- or third-degree burns who developed bacterial infection have been reported. It was confirmed that the wounds did not require split-thickness skin grafts after wound cleansing and treatment with negative pressure therapy. The benefits of NPWT in burn patients, in terms of accelerating wound healing and reducing hospital stay, are also confirmed by the report of the use of NPWT in the treatment of three burn patients in Indonesia [255].

Between 2018 and 2023, 10 articles were published in the PubMed database on clinical trials using NPWT in the treatment of full-thickness and partial-thickness burn wounds [254,255,256,257,258,259,260,261,262,263], 3 of which were dedicated to pediatric burn care [257,259,260].

### 9.3. Hyperbaric Oxygen Therapy (HBOT)

HBOT has shown promising results in improving tissue oxygenation, neovascularization, and reducing inflammation in various clinical scenarios, including burns. It is anticipated that HBOT can lead to faster wound healing, decreased morbidity and mortality in thermal burns and decreased carbon monoxide poisoning [289,290]. Wounds often experience delayed or impaired healing due to chronic hypoxia. The provision of increased oxygen to hypoxic tissues through HBOT addresses this issue and provides significant advantages, especially in the context of wound healing [290]. Some studies demonstrate that incorporating hyperbaric oxygen therapy (HBOT) into a comprehensive approach to burn management resulted in substantial sepsis control among burn patients [291].

However, the current evidence is insufficient to establish the routine use of HBOT in burn care. Conducting large-scale, high-quality multicenter trials is necessary to evaluate the effectiveness, cost, and cost-efficiency of HBOT in burn care, despite the challenges posed by the high variability of burns and chronic wounds [289,290,292]. 

In 2021, Lindenmann et al. [293] provided a comprehensive systematic review of 71 publications from the last twenty years on the molecular mechanisms affected by HBO, taking into account the different medical indications involving tissue repair. They found that only four clinical trials in human patients were available during this period. The prospective randomized trials involved patients with spinal cord injuries and chronic wound healing, such as diabetic foot ulcers. The researchers confirmed the beneficial effects of HBO therapy on wound healing through the stimulation of angiogenesis, the anti-inflammatory effect, the increase in nitrite levels and the stimulation of epidermal and vascular endothelial growth factors [294,295,296,297]. 

The majority of studies on the use of HBO in tissue regeneration are based on experiments using animals, animal or human tissues and cells. The effects of HBO on cytoprotective, anti-inflammatory pathways [298] and factors stimulating differentiation, collagen synthesis, epithelial, neural and angiogenesis have been demonstrated [299]. The analysis of these processes at the molecular level via the quantification of various biomarkers such as vascular endothelial growth factor (VEGF), protein kinase B (Akt), glutathione (GSH), beclin 1 (BCN1), hypoxia inducible factor 1 alpha (HIF1A), transforming growth factor ß (TGF-ß), monocyte chemotactic protein 1 (MCP-1), glycogen synthase kinase glycogen synthase kinase-3 (GSK-3 beta), microtubule-associated proteins 1A/1B light chain 3B (LC3II), mechanistic target of rapamycin (m-ToR), interleukins and others, showed that HBO therapy can alter them in a way that depends on the initial conditions, such as etiology (diabetic, non-diabetic) or level (chronic or acute) of injury [300,301].

Smolle et al. [302] collected papers published in the PubMed database since 1965, i.e., since the beginning of the use of HBO in the treatment of burns. Only 11 papers describing studies in volunteers met the inclusion criteria. The authors of this report point out the wide variety of injuries described and therefore slightly different treatment procedures used (pressure levels, duration of sessions, number of sessions) [303]. However, to ensure the effective downregulation of mediator cascades, HBO therapy ought to be applied as early as possible after the burn injury. In 2021, a literature review on the outcomes of HBOT burn treatment was performed, which showed that the research results to date are controversial [292]. 

Despite the U.S. Food and Drug Administration (FDA) having approved hyperbaric oxygen therapy to help treat, among other things, burns, HBOT can be treated as a supportive treatment (reducing tissue hypoxia and inflammation and supporting neovascularization). 

### 9.4. Platelet-Rich Plasma (PRP)

PRP is a concentrated form of plasma obtained from the patient’s own blood, enriched with platelets through centrifugation. It contains growth factors that aid in tissue regeneration and wound healing through various pathways [304]. PRP also exhibits antibacterial properties and promotes cell proliferation for wound repair. However, the exact mechanism of PRP’s action remains unclear, and there is no standardized preparation method or consensus on its clinical efficacy for burn wound repair [305].

While studies have shown the potential of PRP in treating severe burn wounds and tissue infections, there are limitations to its current systematic evaluation due to the number and quality of included studies. Therefore, further high-quality randomized controlled trials are necessary to establish the efficacy and safety of PRP for burn wound treatment [304,305].

### 9.5. Mesenchymal Stem Cells

Mesenchymal stem cells have a multidirectional differentiation and proliferation capacity that makes them attractive for various medical purposes, particularly in regenerative medicine. Not insignificantly, stem cells can be easily derived from adipose tissue or bone marrow, but also from other tissues such as bone, cartilage, tendon and muscle. The first successful study using cadaveric BM-MSCs was published in 2015 by Mansilla et al. [306]. Human bone marrow-derived MSCs (hBM-MSCs) have regenerative potential and the ability to differentiate into endothelial cells, keratinocyte-like cells and skin appendages [307]. Animal studies have confirmed the usefulness of MSCs in the treatment of burn wounds. MSCs have been shown to secrete a variety of endogenous growth factors and anti-inflammatory cytokines that enable wound healing by promoting angiogenesis, the synthesis of extracellular matrix components, suppressing inflammation and reducing scarring [3,25,308]. Recently, reviews have been published on the use of MSCs in the treatment of burn wounds [309,310,311]. An emerging strategy is the use of stem cells to generate viable autografts. MSCs can be implanted in a special biomaterial construct, which ensures a higher concentration of MSCs at the wound site and increases the secretion of trophic factors.

Stem cell suspensions can be used to print tissues using a method called 3D printing, or bioprinting. The method involves mixing the polymer with a cell suspension and then printing the tissue in a 3D structure. He et al. [312] produced skin replacement scaffolds (TSS) from hBM-MSCs and collagen, which they used to heal wounds on the backs of mice.

The article by Turner et al. [313] describes a three-dimensional core/shell construct that delivers MSCs and endothelial cells for the treatment of thermal skin injury in vitro. The cell lines were encapsulated in gelatin- and chitosan-based capsules. The study observed an increase in the release of wound healing factors, i.e., growth factors, and a reduction in pro-inflammatory factors and signs of neovascularization.

Despite the great potential of MSCs in the treatment of burns and their many preclinical studies, the number of clinical trials is still limited. The first transformation of bone marrow MSCs was performed in 2005 in a patient with burns covering 40% of the body surface [314]. Since then, more than a dozen clinical trials have been conducted on the treatment of burn injuries with MSC-based therapy (www.clinicaltrials.gov, accessed on 29 February 2000).

For example, Xu et al. [315] and Arkoulis et al. [316] used a composite graft consisting of autologous hBM-MSCs embedded in a cell-free allogeneic dermal matrix or autologous hAT-MSCs with a collagen-glycosaminoglycan dermal matrix. However, further research is needed, as the above studies required additional treatment with autografts to ensure adequate efficacy.

There are also no randomized trials or standard procedures for the routine use of MSCs in the treatment of burn wounds, and the available studies have significant differences in methodology.

### 9.6. Growth Factor Therapy (GFT)

Multifunctional polypeptide growth factors (GFs) play an essential role in the healing process of burn wounds by stimulating cell proliferation, angiogenesis and extracellular matrix formation [317]. GFs are also involved in the process of restoring tissue integrity during healing and scar formation. It is known that after burns, the bioavailability of GFs in the wound bed is insufficient [318]. Cloned recombinant GFs have been successfully used to treat chronic wounds such as ulcers and pressure ulcers [319]. The use of GFs in the treatment of burns is described in review articles [320] and in a quantitative meta-analysis of randomized controlled trials (RCTs) by Zhang et al. [212]. The authors demonstrate the superior efficacy of GFs (fibroblast growth factor (FGF), epidermal growth factor (EGF) and granulocyte-macrophage colony-stimulating factor (GM-CSF)) on healing time and scar improvement in terms of pigmentation, pliability, height and vascularity, in partial-thickness burns compared to standard wound care.

Analysis of the data collected shows that the efficacy of GFT depends, among other things, on the method of application. According to Fernández-Montequín et al. [321], the intralesional injection of EGF provides better diffusion and bioavailability than local administration.

In turn, Lee [322] pointed out the possible side effects of both methods, which may not provide an adequate initial concentration of GFs due to the too-rapid degradation and clearance of GFs. The question of the optimal dose of GFs remains a problem. Generally, published reports are characterized by large methodological differences regarding different concentrations of GFs, but also different dressings, antibiotic therapies, etc., making it difficult to assess the effectiveness of GFT treatment.

GFs can also be used in the form of complex preparations, such as recombinant human basic fibroblast growth factor (rh-bFGF) in the local application of insulin (INS) in diabetic patients with deep second-degree burns [323]. Studies showed that the therapy shortened the healing time and reduced pain and the wound exudate rate. An increase in the expression of proteins such as hypoxia-inducible factor-1α (HIF-1α) and vascular endothelial growth factor (VEGF), which promote neovascularization, was also observed. Another randomized study showed the curative effect of rh-bFGF combined with polymyxin B ointment and INS on the wound healing of deep second-degree burns in diabetes mellitus [324].

Since the end of the 20th century, during which Brown [325] used biosynthetic human epidermal growth factor to treat partial-thickness burns, there have been many reports of experiments carried out on animal models using, among others, KGF-1 (FGF-7) [326], vascular EGF (VEGF), (vectors-VEGF165) [327] and basic FGF (bFGF) [328]. The roles of GFs in the wound healing process in burns are described in detail in the review by Miricescu et al. [329].

GFs may provide many benefits in the treatment of burns by accelerating epidermal regeneration, stimulating the proliferation of both epidermis and follicles, accelerating angiogenesis and maturing the extracellular matrix. However, currently, when it comes to the treatment strategy for burn wounds, much more attention is paid to the use of platelet-rich plasma and growth factor-rich plasma instead of the use of biosynthetic GFs [330,331].

## 10. Nutrition/Hypermetabolism

A fundamental aspect of burn treatment involves the importance of appropriate nutrition [18], which should be acknowledged as an integral component within the overall therapeutic process for individuals with burn injuries. When devising a nutritional intervention plan, it is crucial to consider not only the adequate provision of energy and protein but also to carefully evaluate the incorporation of essential macro- and micronutrients [30]. Most articles in the current research agree that a state of hypometabolism occurs during the first 24–48 h post-burn, with a subsequent increase in the metabolic rate until the fifth day post-burn [26,332]. Thus, in the first days after the injury, the patients are in a hypometabolism state [332] with reduced intravascular volume, poor tissue perfusion, low cardiac output, and lower oxygen consumption. Later, there is a phase of hypermetabolism and hypercatabolism, usually in patients with severe burns which can persist for many months (9–12 months) [26]. A burn injury can increase the basal metabolic rate by 50–100% of the normal resting rate. The main features of hypermetabolism include increased oxygen and glucose consumption, insulin resistance, lipolysis, glycogenesis, and muscle protein catabolism [19,333]. Prolonged ICU stays (Intensive Care Unit) in a state of inactivity and chronic catabolism, exacerbate muscle weakness and lean body mass (LBM) depletion [333]. In turn, the loss of LBM affects insulin sensitivity for even up to three years post-burn [333]. 

The hypermetabolic response, and the consequently increased catabolism after burn injury, is a complex process not fully understood, resulting from stress and inflammation [334]. The hypermetabolic reaction is caused by an increase in the concentration of stress hormones, i.e., glucocorticoids (cortisol), the catecholamines epinephrine and norepinephrine, and dopamine, which regulates pancreatic glucagon and insulin secretion via adrenergic and dopaminergic receptors [335]. Inflammatory mediators are also involved in the increase in the post-burn metabolic rate and are responsible for causing systemic inflammatory response syndrome (SIRS) [336]. The immune response to burns is complex [337]. It involves toxic pro-inflammatory mediators and platelet-activating factors released into the circulation. Various chemokines and cytokines are involved in this process, i.e., interleukins (IL), interferons (INF), tumor necrosis factors (TNF), lymphotoxins (LT) and colony-stimulating factors (CSF). Cytokines can generate both pro-inflammatory responses (IL-1, IL-6, TNF-α, IFN-γ) and anti-inflammatory reactions (IL-10, TGF-β). The immune response is associated with the migration to the site of injury of mast cells, neutrophils, dendritic cells, monocytes, macrophages and inflammasomes, which are responsible for regulating the activation of caspase-1 [338], NK and NKT cells, which induce the apoptosis of infected cells. After a burn, complement cascades and C-reactive proteins (CRP), participating in the acute phase response (APR), are induced, which are responsible for the increased risk of SIRS [339].

Two strategies are used to combat post-burn hypermetabolism, i.e., pharmacological and non-pharmacological methods. Early excision and closure of the burn wound can effectively reverse this process. A less frequently used, older strategy is to raise the ambient temperature to as much as 33 °C to reduce the resting energy expenditure of patients in a state of hypermetabolism [71]. 

Another non-pharmacological strategy to combat hypermetabolism is appropriate enteral nutrition [340]. Burns contribute to altered glucose, protein and lipid metabolism and increased energy expenditure. For burns below 20% of the TBSA, oral calorie intake is usually adequate. However, for larger burns or patients needing prolonged intubation, the early placement of an enteral feeding tube is recommended [10]. Parenteral nutrition should be used selectively for specific conditions like paralytic ileus, pancreatitis, bowel obstruction, or contraindications to enteral feeding, as it is associated with higher infection rates due to prolonged central venous access requirements [26]. Preventing weight loss exceeding 10% of the baseline is crucial for better outcomes. A loss of lean body mass (LBM) beyond 10% can impair immune function and delay wound healing. Reductions exceeding 40% of the LBM are life-threatening [19]. To date, various formulae have been developed to estimate caloric requirements and calculate resting energy expenditure (REE) for adult patients hospitalized for burns, i.e., the Curreri formula, Harris–Benedict, Toronto formula, Davies and Lilijedahl, Ireton-Jones, and for children, i.e., Galveston, Curreri junior [341,342].

Formulae usually need to be adjusted according to changes in monitored parameters, as the energy expenditure will be different during periods of peak energy expenditure and at the end of treatment. Therefore, there is a risk of malnutrition and the overestimation of the caloric requirements of burn patients.

In a 2002 study [343], the accuracy of up to 46 REE estimation methods was evaluated in 46 patients with greater than or equal to 20% of their TBSA burned after thermal injury. The authors considered the methods of Milner (1994), Zawacki (1970) and Xie (1993) to be the most accurate. 

Patients with burns should receive low-fat food rich in carbohydrates and amino acids. Additional supplementation is under examination. There are many studies focusing on trace element imbalances in burnt patients such as copper, selenium and zinc, as they have a great role in wound healing, the immune system and the anti-oxidant system, and are easily lost in burn exudates [6]. Antioxidant therapy in burns should supply ascorbic acid involved in the synthesis of collagen, glutathione, carotenoids and vitamin A, important for immune function and epithelialization, vitamin E, calcium and vitamin D, the usefulness of which have not yet been determined in burned subjects [344,345]. In 2022, the first double-blind, randomized, controlled multicenter clinical trial (RE-ENERGIZE) was conducted on 1209 patients with severe burns (an average of 33% of their total body surface area), regarding glutamine supplementation in the treatment of severe burns [346]. The study group received 0.5 g/kg body weight per day of glutamine enterally. The authors found that this strategy had no effect on recovery rate or mortality. In an earlier study from 2006 [347], it was proven that glutamine may be potentially ‘life-saving’ in critical illnesses, particularly when administered in doses greater than 0.3 g/kg/day. Lin et al. [348], in a meta-analysis of double-blind randomized clinical trials, in a group of 155 people proved that the supplementation used did not affect the duration of treatment or the risk of wound infection, but it did reduce mortality and reduced the risk of blood infection with Gram-negative bacteria. Similar results regarding glutamine supplementation were obtained in a meta-analysis by other authors [349]. Arginine also has immunomodulatory properties. Yan et al. [350] showed that enteral nutrition with the addition of l-arginine at the early stage of burn reduces NO production and improves blood supply and tissue oxygenation. In order to strengthen the immune response in burnt patients, several immunomodulatory additives are most often applied, such as omega-3 fatty acids (fish oils), especially eicosapentaenoic acid (EPA) and docosahexaenoic acid (DHA), as well as glutamate and arginine. In a 2009 paper, Marín et al. showed that the addition of 30% omega-3 fatty acids (including 20% EPA and 10% DHA) to the diet reverses unfavorable changes in the plasma lipidome, promoting protein metabolism and modulating the immune response in burned children [351]. Taking increased amounts of omega-3 fatty acids reduced the risk of sepsis and non-infectious complications in adult patients with burns covering 38% of their body surface [352]. The beneficial effects of omega-3 are responsible for their so-called derivatives, resolvins, which are produced in neutrophils during the inflammatory process. They are believed to have many beneficial effects, such as inhibiting the secretion of IL-1β and TNF-α, preventing the formation of clots in the vascular layer of the skin, having a beneficial effect on blood supply, increasing the migration of neutrophils to the site of injury and having anti-inflammatory effects [353]. In an animal model of severe burns, the nephro- and hepatoprotective effect of resolvins was proven [354].

Currently, applied pharmacological modulators of the post-burn hypermetabolic response include recombinant human growth hormone (rhGH), a synthetic testosterone analog (oxandrolone), a beta-blocker drug and a low dose of insulin. 

The increase in catecholamine levels after a burn correlates with the level of metabolism and plays a key role in changing the fat phenotype [355,356,357]. It is understandable that the beta-blocker drug propranolol reduces burn-induced hypermetabolic catabolism [358]. It turns out that this non-selective β1/β2 receptor antagonist, in addition to reversing catabolic reactions in the form of skeletal muscle atrophy and lean body mass, contributes to reducing insulin resistance, which is typical of a hypermetabolic reaction [359]. The beneficial effects of propranolol were confirmed in a prospective, randomized, single-center, controlled study in pediatric patients with large burns [358], on one-hundred seventy-nine pediatric patients with more than 30% total body surface area burns. The authors confirmed that propranolol treatment for 12 months after thermal injury ameliorated the hyperdynamic, hypermetabolic, hypercatabolic and osteopenic responses in pediatric patients.

The post-burn hypermetabolic response can be also minimized via the infusion of a low dose of insulin, which allows for control of the hyperglycemia caused by burns. Many studies confirm that insulin therapy reduces the incidence of infections in burn patients [360,361,362]. The occurrence of hyperglycemia is probably related to endoplasmic reticulum (ER) stress in damaged tissues. It has been established that the reaction is mediated by the NLRP3 inflammasome, present in adipose tissue [363]. Possible, dangerous and poor prognosis episodes of hypoglycemia [364,365] require strict glycemic control to the optimal value of 130 mg/dL. A much safer option, in comparison to insulin, appears to be biguanides such as metformin, or other antidiabetic drugs, such as (GLP)-1, pioglitazone, thioglitazones and fenofibrate [366,367].

In turn, RhGH administration is not currently recommended because, despite its anabolic effect, it causes hyperglycemia and insulin resistance [368]. Better results have been associated with insulin-like growth factor (IGF-I), which in animal studies showed beneficial anti-inflammatory and anabolic effects. The complex developed so far, IGF-I with binding protein (IGFBP-3), did not induce hyperglycemia and proved beneficial in the treatment of pediatric patients [369]. The tests were performed on nine children with burns covering >40% of their TBSA. The authors claim that low-dose IGF-1/BP-3 effectively attenuated the type I and type II hepatic acute phase response, increased the serum levels of constitutive proteins and modulated the hypermetabolic response.

Another anabolic drug is oxandrolone. There are many years of positive experience with the use of this synthetic testosterone analog in the treatment of burn patients [370]. Oxandrolone was introduced for medical use in the 1960s. It is a synthetic 17-alpha-methyl derivative of testosterone. Its anabolic activity is about 10 times greater than that of original testosterone, with minimal androgenic side effects. Oxandrolone has been used in people following severe burns. A prospective cohort study of 14 severely burned children with greater than 20% of their TBSA burned, and nutrient depletion, admitted to the Shriners Burns Children’s Hospital in Galveston, Texas, evaluated the efficacy of treatment with oxandrolone 0.1 mg/kg orally, twice daily, compared with a control group without pharmacological treatment [371]. As a result of the study, an improvement was observed in the oxandrolone group, which was associated with the increased efficiency of protein synthesis. Due to conflicting reports on the side effects of this drug, Ring et al. [372] performed a systematic review of 31 randomized controlled and observational studies on the effect of oxandrolone on the treatment of burn patients. The authors searched PubMed, EMBASE, Web of Science, CINAHL and Cochrane databases. The study found that oxandrolone had a positive effect on shortening hospital stays and improving several parameters of growth and wound healing. The authors did not agree that oxandrolone increased the risk of liver damage (relative risk RR: 1.04; 95% CI: 0.59 to 1.85; *p* = 0.88). Similar conclusions were reached in another systematic review and meta-analysis of 15 randomized controlled trials involving 806 patients, by Li et al. [373], on the safety of oxandrolone in the treatment of patients with severe burns. Oxandrolone therapy did not show a significant difference in liver dysfunction (RR = 1.15, (0.83, 1.59), *p* = 0.41). Oxandrolone treatment had a positive effect as it shortened hospitalization time, healing time and time between treatments, prevented surgical procedures, weight loss and nitrogen loss. The long-term effect was an observed increase in lean body mass of approximately 4% after half a year and approximately 10% after one year (*p* < 0.00001). Another systematic review compared the efficacy of oxandrolone in the treatment of patients with burns to patients with pressure ulcers. Double-blind, randomized trials demonstrated the beneficial effect of oxandrolone on wound healing [374]. In a study of 52 burn patients with more than 40% of their TBSA burned, the effect of oxandrolone on mortality and sepsis was investigated [375]. The efficacy of oxandrolone was also evaluated in a population of 61 children with burns on 40% of their TBSA treated for one year [376].

In that study, the authors reported beneficial effects of the oxandrolone therapy compared with the control group, i.e., an improvement in lean body mass, improvement in bone mineralization and improvement in muscle strength (*p* < 0.05). The authors concluded that the use of oxandrolone significantly reduced mortality within 28 days (OR 0.11, 95% CI: 0.04–0.30) and the occurrence of sepsis (OR 0.24, 95% CI: 0.08–0.69). However, the authors point out that oxandrolone increased the risk of multiple organ failure (MOF) (OR 7.90, 95% CI: 2.89–21.60). There are also reports indicating that oxandrolone may cause liver damage [377] or acute respiratory distress syndrome in ventilated patients, and delayed wound healing [378].

On 28 June 2023, the FDA withdrew the drug and its generic versions from medical use in the United States. Since then, oxandrolone has no longer been used to mitigate post-burn hypermetabolism. Instead, some burn centers use another anabolic steroid, nandrolone decanoate (ND). A prospective randomized control study from 2022, which included 40 patients burned on 20–40% of their TBSA, confirmed the effectiveness of ND in combating catabolic symptoms in burn patients, manifested in the form of improved nitrogen balance and the preservation of lean body mass [379]. 

## 11. Pain Management

Burn patients experience pain due to irritation of the nerve endings exposed by damaged tissue. Pain in burns is due to the damage and treatments such as dressing changes and skin grafting [4,26,380,381]. While first- and second-degree burns are very painful, third-degree burns are usually painless because the pain receptors in the skin have been damaged. However, nociceptors around the coagulation zone can still transmit pain impulses. Difficulties in nerve regeneration in burn patients can lead to neuropathic pain [381]. It is said that the complete reorganization of nerve fibers can take up to 6 months after injury. Effective pain management is essential to prevent complications such as hyperalgesia [79], allodynia and depression [4]. The surgical or enzymatic debridement of burn wounds followed by grafting or appropriate dressing can reduce pain [26].

Burn pain is complex and dynamic, often evolving as patients undergo multiple procedures and treatments involving the manipulation of their burn sites. However, despite the recognition of the importance of pain management in burn wound healing, there are numerous reports highlighting the inadequacy of burn pain management [381].

**Figure 5:**
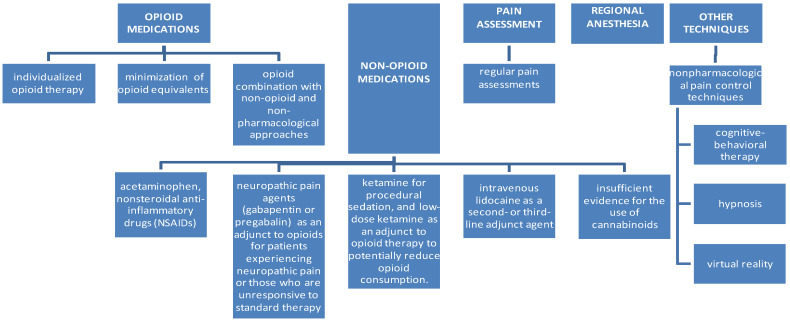
The guidelines of pain management.

Pain management in burn patients is a very complex issue and should be individualized [27], taking into account the wide range of pharmacological treatments available [368]. Some of the guidelines for pain management are illustrated in Figure 5. 

Pain management in young children is a major challenge, given that more than half of all burns occur in children under the age of 5 years [382]. The chronic exposure to severe pain associated with burn care can lead to permanent changes in neuronal plasticity in the brain and the development of PTSD [383,384]. The use of opioids does not have the desired effect in almost 50% of children [385]. In addition, anesthesia and sedation are associated with the possibility of behavioral, emotional and cognitive dysfunction [386,387,388]. Therefore, there is a growing interest in non-pharmacological pain management therapies [389,390]. Entertainment or immersive virtual reality (VR) has been found to be effective in children under 6 years of age [391]. Virtual reality can effectively distract attention and reduce acute pain [392,393].

In a recent study, the analgesic efficacy of a desktop VR system (without a VR helmet) was measured in children under six years of age during burn wound dressing [394]. Nine children with burns (aged 10 months to 5 years, mean = 18 months) participated in the study. Pain was assessed using the Faces, Legs, Activity, Crying and Consolability (FLACC) pain scale. Observational results showed that the combination of desktop virtual reality and traditional analgesics reduced anxiety by more than 30% compared to pharmacological treatment alone (*p* < 0.005).

Recently, the same positive effect has been observed in young children under the age of 6 [395,396]. In this study, the image was displayed on an immersive dome screen. An fMRI study confirmed that VR can reduce pain-related brain activity and subjective pain to a degree comparable to a moderate dose of hydromorphone [397].

A study from 2021 [398] identified plastic changes in the brain following different types of burns. The aim of the study was to map cerebral blood volume (CBV) in different brain regions (postcentral gyrus, frontal lobe, temporal lobe and insula) using MRI images and SPM12 software (The Wellcome Centre for Human Neuroimaging, UCL Queen Square Institute of Neurology, London, UK; https://www.fil.ion.ucl.ac.uk/spm/software/spm12/, accessed on 1 October 2014). The study included 60 people with electrical (EB) and non-electrical (NEB) burns and 20 patients in the control group. It appeared that there was no difference in CBV between the two groups of burn patients, but in the EB group with chronic burn pain, CBV decreased significantly in brain areas on the same side as the burn. There were changes in the frontal and temporal lobes. The study showed the following changes: an increase in CBV in the postcentral gyrus and a decrease in the temporal lobe and insular cortex, frontal lobe and precentral gyrus. The authors concluded that chronic burn-induced pain causes plastic changes in the pain network that are limited to the sensory-discriminative dimension. 

Despite the availability of alternative treatments, opioids remain the most effective drugs for post-burn analgesia. Opioids are usually prescribed at higher doses and for longer periods of time, despite the risk of addiction and the CDC recommendations to minimize the dose and duration of opioid treatment [399,400,401,402]. Patients with burns experience many types of pain, including procedural, background and breakthrough pain. Pain can be localized at the site of injury (primary pain) or elsewhere (secondary pain). The long-term consequence of burn injury is the development of hyperalgesia, including chronic neuropathic pain, due to central sensitization and alterations in intracellular signaling pathways [403,404].

Background pain is treated with opioids, i.e., morphine, oxycodone and methadone with moderate potency and a long half-life. Procedural pain is treated with continuous short-acting high-potency opioids, i.e., fentanyl, alfentanil or remifentanil. Breakthrough pain is treated with intravenous morphine or intranasal or buccal fentanyl, e.g., in the form of lozenges for children. Attention should be paid to individual variability in the metabolism of some opioids, such as methadone, which may increase the risk of overdose [405,406,407].

Opioids have the ability to block pain signals by activating three types of receptors: m (µ)-, kappa (k)- and delta (d)-opioid receptors. Conventional opioids are m-selective, but their activation has many negative side effects in addition to analgesia. Research is underway to develop analgesics specific to d and k receptors, which would eliminate the effects of opioid agonism, i.e., respiratory depression and addiction. These studies have shown that women have significantly greater k-opioid receptor-mediated analgesia than men [408,409,410]. All opioid receptors are G-protein-coupled receptors (GPCRs) [411]. Opioid receptors can also signal via the G-protein-independent mechanisms [412] of β-arrestin, GRK isoforms, the MAPK family [413] and intracellular signaling pathways [414]. Therefore, opioid agonists can induce different signaling effects in the cell [415]. Signaling via the G-protein-activated pathway enhances analgesia, whereas the β-Arr2-dependent pathway mediates the development of adverse effects such as respiratory depression and the development of opioid tolerance [416]. There is also evidence for the involvement of non-neural mechanisms in the antinociceptive effects of opioids [417]. For example, opioids have been shown to activate Toll-like receptors (TLRs) on immune cells [418]. Chronic opioid use is unfavorable not only because of the development of antinociceptive tolerance, but also because of effects such as hyperalgesia and allodynia [419].

There is ample evidence that burn injury reduces the antinociceptive effect of opioids [420,421] as a result of changes in the functionality of the immune system, the development of inflammation [18] and even the so-called systemic inflammatory response syndrome (SIRS) [422], as well as changes in the expression of receptor, effector and signaling molecules. Burns alter the expression of m-opioid receptors in the dorsal horn of the spinal cord [423] and the NMDA receptor, as well as molecules such as Akt, protein kinase C, nitric oxide synthase and glycogen synthase kinase 3b. Burn-induced changes in the NMDA receptor reduce the analgesic efficacy of both opioids and ketamine [399]. The development of antinociceptive tolerance to opioids is also the result of changes in Akt/mTOR, p38-MAPK and JNK signaling [424,425] and effector molecules such as b-arrestin [416]. In animal studies [420], burn injury has been shown to reduce the potency of opioids, and antinociceptive tolerance is more likely to respond to treatment with morphine than to treatment with oxycodone or hydrocodone. Interestingly, the effect was seen in adult rats, but not juvenile rats [426]. A study in mice [427,428] showed that burn injury caused a greater reduction in the antinociceptive response to opioids (morphine, oxycodone and hydrocodone) in the contralateral limb than in the burned limb. It is likely that the effects of opioids are antagonized by inflammatory signals in the damaged tissue.

Opioids have variable efficacy depending on the pain phenotype in animals and humans [429]. For example, oxycodone and morphine partially reduce allodynia but do not prevent the development of hyperalgesia caused by burns. Morphine treatment can exacerbate and prolong neuropathic pain in rats, even after cessation of treatment [430]. Hydrocodone has been shown to be effective in relieving burn-induced hyperalgesia and reversing mechanical allodynia [431].

## 12. Psychological Advisory

As survival rates improve, the focus of burn injury outcomes has shifted toward evaluating functional abilities and integration into the community. Burn injuries can have a profound impact, causing lasting physical and emotional consequences. Deep burns leave disfiguring scars, which can significantly change a person’s appearance and require them to cope with body image alterations. Furthermore, the traumatic nature of the burn incident and the painful treatments involved can trigger psychological reactions. Studies have observed that severely burned adult patients generally exhibit good adaptation, although some may experience notable psychological disturbances such as somatization and phobic anxiety. Among burn patients, depression and PTSD have been extensively studied, with prevalence rates ranging from 13% to 23% and 13% to 45%, respectively [7,15]. 

Considering the finding that a notable portion of burn patients experience psychological issues, which greatly disrupt their everyday lives, it is essential to identify high-risk patients and provide them access to psychological treatment [15].

Severe burns have a psychological impact. About 30% of patients experience symptoms of PTSD. Patients with facial burns are particularly at risk [432]. A 2017 study by Bond et al. [433] also found high rates of PTSD in the close relatives of burn patients. The authors of the study emphasize that depressive symptoms are more strongly correlated with self-report scores than with the actual severity of the injury. In addition to mental health, other consequences of burns that require treatment include pruritus [404], chronic pain and neuropathy [434].

A prospective matched cohort study conducted at a burn treatment center in Germany in 2023 examined the impact of facial burns on short- and long-term quality of life and psychological distress [435]. The study included 55 patients with facial burns (FB) and 55 controls. Cases of fire/flame, electrical, scalding and contact burns were included in the study group. The study groups were assessed using the 36-item Short Form (SF-36) and the Hospital Anxiety and Depression Scale (HADS). They found that even 1 year after injury, the FB group had worse scores on the physical and mental dimensions and higher scores for anxiety (*p* ≤ 0.002) and depression (*p* = 0.01) compared to the FB control group. At 1 year post injury, there was a trend towards improvement in physical functioning (*p* = 0.02) and body pain (*p* = 0.01). An observational cross-sectional study conducted in the Departments of Psychiatry and Burns and Plastic Surgery at the All India Institute of Medical Sciences, Rishikesh, analyzed the psychological consequences of burns in groups of male and female patients [436].

The study included 32 patients who were assessed for psychiatric diagnosis, severity of depression and anxiety, quality of life and scar scores. The study showed no significant differences in the severity of anxiety or depression between the sexes. When it comes to the experiences of Pakistani women with facial burns, some differences in attribution style and post-injury perception have been observed [437]. People with high self-esteem, strong family support and available resources have a positive outlook on life and find it easier to accept changes in their appearance after an injury. In a long-term study, 5, 10, 15 and 20 years after the burn, the effects of the injury on health, i.e., functional and psychological components, were evaluated by analyzing data from the Burn Model System National Longitudinal Database (1993–2020) [438]. The study included 421 adults who had been burnt. Mental deterioration was associated with a longer hospital stay, the female sex and a longer time since injury. Worse social integration was also observed in relation to Hispanic/Latino ethnicity. The severity of a burn affected the mental state of burn patients. In a cross-sectional study of 225 patients hospitalized in Pakistan in 2019–2020, variation in the level of anxiety was observed in relation to the depth of the burn [439]. Most patients with superficial burns had mild anxiety (69.5%) and only 3.8% had severe anxiety. In deep burns, 45.3% of patients experienced severe depression. Patients with severe burns who undergo amputation face the greatest psychological challenges.

A retrospective cohort study based on data from the Burn Centre of Adana City Training and Research Hospital (ACTRH) from 2016 to 2020 shows that the frequency of amputation is not high, at 1.9% of burns [440].

## 13. Rehabilitation and Scar Treatment during and after the Acute Phase

Programs for physical and occupational therapy in burn injuries prioritize fast mobilization [19] for the restoration and preservation of a range of motion, scar reduction and the prevention of contractures [79]. The mobilization should start as early as possible, even during the ICU stay [27]. However, it is important to also consider the potential physical and functional limitations that can hinder individuals with burn injuries from achieving independence [333].

Deep burns with a prolonged inflammatory phase can lead to the development of pathological scars, i.e., hypertrophic scars and keloids. Pathological scars are characterized by excessive bundles of cross-linked collagen due to reduced collagenase activity. Scars are not only unsightly but also cause contractures that require physical rehabilitation [441]. In hypertrophic scars, the expression of type I collagen is reduced and type III collagen is over-synthesized [442]. Hypertrophic scarring is a potential complication [23,24] that can occur in grafted wounds and unexcised wounds that take longer than 2 to 3 weeks to heal. Patients with pigmented skin have a higher risk of developing hypertrophic scarring. On the other hand, keloid scars are dark, raised, fibroproliferative lesions of a neoplastic nature [443], consisting of a bundle of type I and type III collagen [441]. An extensive review on the biology, prophylaxis and treatment strategies for hypertrophic scars and keloids has recently been published in *Int. J. Mol. Sci.* [444]. There are various strategies available to prevent [441] or reduce the severity of hypertrophic scarring and keloids. 

Pressure garments are commonly used on grafted or slow-healing areas, as the elastic support provided by these garments can alleviate symptoms such as throbbing and itching [445], however, the underlying mechanism of action, involving decreased collagen synthesis in scar tissue due to limitations in the supply of blood, oxygen and nutrients [445,446], as well as increased apoptosis [447], is still being discussed [7,448].

Silicone has also been recommended for the treatment [449] and prevention [441] of hypertrophic scarring. Steroid injections have been used to alleviate symptoms associated with hypertrophic scarring. The therapeutic effect is most likely attributed to occlusion and hydration mechanisms [441]. Corticosteroids primarily exert their effects by suppressing the inflammatory process in the wound [441,450]. They also have secondary effects, including the reduced synthesis of collagen85 and glycosaminoglycans, and the inhibition of fibroblast growth [7,450].

Cryotherapy has been used alone or in combination with other treatments for excessive scars. When combined with steroid injections [446], cryotherapy has shown significant improvement in hypertrophic scars and keloids. It is believed that cryotherapy induces vascular damage, leading to anoxia and tissue necrosis. However, its effectiveness is limited to treating small scars [441]. Nevertheless, the scientific evidence supporting the efficacy of these techniques varies, and further research is needed to establish their effectiveness [26,450].

In current treatments, due to laser therapy, the direct reduction of scar volume at least 1 year after the primary wound treatment, in combination with radiotherapy adjuvant 1–2 days after scar revision, can be achieved, ensuring angiogenesis and inflammation inhibition. A similar effect can be achieved using an intralesional injection of 5-fluorouracil.

The use of mesenchymal stem cells (MSCs) for the treatment of hypertrophic scars and keloids requires further research. Although many beneficial effects have been attributed to MSCs [451], such as inhibiting the activity of pro-inflammatory cells, reducing collagen type I and III production and promoting angiogenesis, there are also conflicting reports [452].

Trials of autologous fat grafting, which is also a source of MSCs [453], and combination therapy of interferon IFN α-2b with injection of triamcinolone acetonide TAC [454] also showed beneficial effects. Intralesional injections of botulinum toxin [455] and bleomycin were beneficial in 54% to 73% of cases of keloids [456]. Clinical trials using recombinant human transforming growth factor-β (TGF-β3) to reduce fibrosis have been unsuccessful [457].

To minimize or prevent hypertrophic scars and keloids, consideration should be given to mechanotransduction processes [458], which impose high mechanical stresses on the wound with a tendency towards poor scar formation. Therefore, tension-free wound closure and passive mechanical stabilization are preferred [459]. The efficacy of preparations containing substances of plant origin, i.e., Contractubex and Mederma Skin Care Gel, containing allium cepa extract and allantoin, is controversial [460].

Similar to post-operative skin wound compression, despite some clinical experience and indications, efficacy in the treatment of hypertrophic scars and keloids is poorly documented [461].

The type of dressing used is also important in preventing scarring. A clinical trial conducted in 2022 compared Suprathel^®^ with Dressilk^®^ [462]. Suprathel^®^ is an example of a biosynthetic copolymer for the treatment of second-degree superficial burns, and Dressilk^®^ is a silk dressing made from protein fibroin. In a group of 12 patients, wound healing and scar quality were compared after treatment with the two dressings. The authors of the study recommend both dressings based on their clinical experience at the University of Witten-Herdecke and the University Hospital Witten-Herdecke in Germany. However, they point to the economic aspect and the fact that wounds treated with Dressilk^®^ showed a faster return to intact skin quality and significantly higher oxygen saturation in the wound areas (*p* = 0.008).

## 14. Systemic Antibiotic Therapy

Burn patients are vulnerable to infections from multiple origins, and their burn wounds rapidly become colonized by microorganisms od endogenous and exogenous origin, especially Gram-positive bacteria, such as Staphylococci, which are often present in the exposed sweat glands and hair follicles [463]. Although antibiotic prophylaxis reduces mortality in intensive care patients, it is rarely used for burns due to concerns about eliminating normal flora and promoting antibiotic resistance, and an increased risk of opportunistic infection [26]. In cases where patients have contaminated wounds, are immunocompromised, or undergoing surgery, it is advisable to determine the target microorganisms with consideration of the facility and local characteristics [7,39]. This should be carried out by examining bacterial cultures from the wound. As an option, the preventive systemic administration of antibiotics may be considered [39].

However, current guidelines do not recommend systemic antibiotic prophylaxis for burn patients due to insufficient evidence of their effectiveness and the risk of antibiotic resistance [19,39,463]. 

Furthermore, since burn eschar lacks microcirculation, there is no way for systemically administered antibiotics to reach the affected area. Hence, it is necessary for topical agents to offer broad-spectrum antimicrobial coverage directly at the colonization site [26].

Antibiotics and antifungals should be reserved for patients who show systemic signs of sepsis. It is also important to keep in mind that burn patients are at a high risk of developing ventilator-associated pneumonia and central line-associated infections [7]. Sepsis develops usually several weeks after a burn injury [464]. Much progress has been made in understanding the immunopathogenesis of burns and disorders of innate and adaptive immunity. Immunobiochemical markers predictive of sepsis, such as cytokines, growth factors, C-reactive protein, procalcitonin, presepsin, matrix metalloproteinases, reactive oxygen species, nitric oxide and hemostasis parameters, should be monitored to allow the timely initiation of a specific therapy [465]. However, traditional indicators of sepsis show poor performance, mainly due to hypermetabolic and inflammatory reactions after burns. Sepsis has been predicted with 100% accuracy at initial surgery using the flow cytometry analysis of body fat [466]. However, these results should be treated with caution as only 37 patients were included in the study. Genomic and proteomic changes after burn injury are being systematically collected as part of the Glue Grant project [68,467]. Tran et al. [468] developed an automated machine learning platform (Machine Intelligence Learning Optimizer, MILO) for the prediction of burn sepsis based on the analysis of 211 adult patients with severe burns. Seven predictors of burn sepsis were identified, controlling for age and burn size (OR 2.8, 95% CI 1.99–4.04, *p* = 0.032). The optimal model used 16 of the 23 characteristics tested, i.e., mean arterial pressure, respiratory rate, body temperature, Glascow Coma Score (GCS), white blood cell count (WBC), hemoglobin (HGB), hematocrit (HCT), platelet count (PLT), sodium (Na^+^), potassium (K^+^), blood urea nitrogen (BUN), plasma creatinine, glucose, total carbon dioxide (TCO_2_) and multiple organ dysfunction score (MODS). The accuracy of the model was 86% with an receiver operator characteristic (ROC)—the area under the curve (AUC)—of 0.96. The authors of the study found it interesting that PLT, which is one of the criteria for burn sepsis, was excluded from the algorithm, while HGB and TCO_2_ were included. This is justified because sepsis is associated with hematological and acid-base and electrolyte abnormalities, and PLT is an important criterion for late-stage burn sepsis.

## 15. Conclusions and Future Directions

Technological advances in recent years have made burn care more effective, resulting in a reduction in burn deaths. Most progress has been made in the field of biomaterials and tissue engineering, but an ideal biomaterial that mimics the structure of the skin and is able to restore skin function, pigmentation, skin appendages, vessels and nerves, has not yet been developed. Randomized trials in large populations are still needed to evaluate the effectiveness of experimental therapies using new dressings and skin substitutes. Acellular and cellular tissue-engineered skin constructs appear to be promising. One of the current research trends is the attempt to create a skin substitute as a result of the rapid cultivation of stem cells on special polymeric substrates, which would cover wounds, but also as bioactive dressings that actively support the functionality of the wound and accelerate the healing process. There is also a lack of clinical trials on the use of cytokine growth factors in humans to improve burn wound healing, although there is ample evidence of their efficacy (PDGF, FGB, EGF, TGF-α, VEGF, IGF-I, NGF, TGF-β, GM-CSF and ACCS). Strategies to regenerate and restore the thermoregulatory function of damaged sweat glands are also needed. Further research is also needed on the possibility of reducing the metabolic rate after burns through appropriate complementary nutritional and pharmacological strategies.

## Figures and Tables

**Table 1:** Classification of burns.

Feature/ Degree	I Superficial	IIA Superficial Partial Thickness	IIB Deep Partial Thickness	III Full Thickness	IV Full Thickness and Tissues Beneath
depth of the wound	epidermis only	epidermis + papillary dermis (saving skin appendages)	epidermis + papillary dermis + reticular dermis	epidermis + dermis + hypodermis	epidermis + dermis + hypodermis + tissues beneath (e.g., fascia, bones, tendons)
counted in the calculation of TBSA	NO	YES	YES	YES	YES
common cause	sunburn	scald (short exposure)	scald, oil (medium time exposure)	scald (long exposure), flame, steam, oil, chemical compounds, electricity	scald (long exposure), flame, steam, oil, chemical compounds, electricity
characteristics	swelling, erythema, dryness, blanches with pressure	blisters filled with serum (as a result of the delamination of the epidermis from the basement membrane), blanches with pressure	blisters filled with serum, variety of colors: white/red/grey/yellow, usually excised and grafted	hard, waxy, leathery consistency, color: white/gray/charred, does not bleed when scratched, surgical treatment obligatory	hard, waxy, leathery consistency, color: white/gray/charred, does not bleed when scratched, reaching deeper structures, surgical treatment obligatory
sensation	painful	extremely painful	painful or decreased sensation	insensate	insensate
healing time	subsides after 72 h, skin peels off after 10 days, no scarring	up to 3 weeks, possible color match defect, hypertrophic scarring (low to moderate risk)	up to 9 weeks, hypertrophic scarring (moderate to high risk)	no healing capacity, without intervention it will separate over the weeks, hypertrophic scarring (high risk)	no healing capacity
measures	should heal by itself	should heal by itself	surgical intervention	surgical intervention	surgical intervention

**Table 2:** Common dressings used in superficial partial-thickness burn injury management [116,125,126,127,128,129].

Dressings	Category	Dressing Material/Antimicrobial Agents
Urgosorb^®^ Silver, Urgotul SSD, Urgotul^®^ Silver, Urgotul Duo Silver, Allevyn^®^ Ag Gentle	Antimicrobial silver dressings, soft polymer	polyester mesh impregnated with hydrocolloid, petroleum jelly/silver sulfadiazine (SSD)
Bactigras^®^	Low-adherence wound-dressing pads	white soft paraffin tulle dressing/chlorhexidine acetate BP
Acticoat^®^, Atrauman^®^ Ag	Low-adherence antimicrobial silver dressings	high-density polyethylene mesh of a rayon-polyester with nanocrystalline silver
Algosteril, Comfeel Alginate Dressing, Carrasorb H, Kaltostat, Cardinal Health™ Reinforced Gelling Fibers Algisite M	Fiber dressings, alginates	calcium alginate dressing derived from seaweed
Askina Calgitrol Ag^®^, Algicell^®^ Ag, Tegaderm^®^ Aliginate Ag, Sorbsan Silver Flat, Silvercel^®^, Melgisorb^®^ Ag, Biatain Alginate Ag, Acticoat^®^ Absorbent, Silvercel^®^, Algisite^®^ Ag, Suprasorb^®^ A + Ag, Actisorb^®^22	Antimicrobial silver dressings, alginates	silver alginate wound dressing consisting of an absorbent foam sheet coated with an alginate matrix containing ionic silver and a superabsorbent starch co-polymer
OpSite, Tegaderm	Film/membrane dressings	adhesive-coated hydrophilic polyurethane film
Aquacel Ag^®^ Physiotulle^®^ Ag	Antimicrobial silver dressings, hydrofibers	sodium carboxymethylcellulose fibers containing 1.2% ionic silver
Comfeel, DuoDerm, Comfeel	Hydrocolloid dressings	hydrocolloids (gelatin, pectin and sodium carboxymethylcellulose) in an adhesive polymer matrix
Aqua clear, Nu-gel, IntraSite, Aqua-Gel^®^, Burnaid^®^, Water-Jel, Hydrosorb	Hydrogel dressings	amorphous hydrogels; high-water-content gels containing insoluble polymers (modified carboxymethylcellulose, hemicellulose, agar, glycerol, alginate, collagen, chitosan and pectin) or synthetic polymers such as polyvinyl alcohol or polyethylene glycol
Kerracel™ Gelling Fiber Dressing, AGILE™, AQUACEL^®^ EXTRA™ Hydrofiber^®^ Wound Dressing with Strengthening Fiber	Hydrofiber dressings	sodium carboxymethyl cellulose, strengthening cellulose fibers that form a gel when in contact with exudate
Mepitel^®^	Silicon-coated nylon dressings, low-adherent dressings, foams	safetac layer, made of soft silicone and porous, transparent and flexible polyamide mesh
Actisorb^®^ Silver	Odor absorbent antimicrobial silver dressings	activated charcoal dressing with silver, enclosed in a non-adherent nylon sleeve
Biobrane, TransCyte, AWBAT-S	Biosynthetic skin substitute dressings	cyanuric chloride and dodecylamine covalently bond the collagen peptide to the silicone–nylon composite
Mepilex Border Ag, Mepilex Ag, Allevyn, Biatain	Foam dressings	polyurethane foam dressing with silver and activated carbon with a Safetac silicone contact layer
Suprathel	Synthetic antibacterial dressing, polylactic membranes (PLM)	copolymer of DL-lactide (>70%) and ε-caprolactone
Silverlon@ Wound Contact, Burn Contact Dressings	Dressing pads	Silverlon knitted nylon material plated with 99% elemental silver and 1% silver oxide
PermeaDerm, PermeaDerm B, PermeaDerm CW, PermeaDerm T, PermeaDerm AS	Adherent dressing, a synthetic matrix	a monofilament nylon knitted fabric, bonded to a silicone membrane (B, CW), coated with a mixture of porcine gelatin and a fraction of aloe vera or HMW-native collagen (T) or antiscar coating (AS)
PuraPly^®^ Antimicrobial (PuraPly AM) Wound Matrix	Antimicrobial dressing, cross-linked extracellular matrix	a collagen sheet/0.1% polyhexmethylenebiguanide hydrochloride

**Table 3:** Phytochemical-based nano-pharmacotherapeutics.

Plant	Active Components	Nanocarrier Type	Nanocarrier Component	Refs.
*Rheum officinale*	emodin (1,3,8-trihydroxy-6-methyl-anthraquinone)	nanofibers	polyvinylpyrrolidone	[181,182]
*Polygonum cuspidatum*	emodin	nanofibers	ultra-fine cellulose acetate	[183]
*Centella asiatica*	asiaticoside	nanofibers	trisachharide triterpene and cellulose acetate	[184]
*Curcuma longa*	1,7-bis(4-hydroxy-3-methoxyphenyl)-1,6-heptadiene-3,5-dione	nanofibers	cellulose acetate	[185]
*cinnamon, lemongrass,* *peppermint*	essential oils	nanofibers	cellulose-based nanofibers	[186]
*Curcuma longa*	curcumin	nanofibers	poly(ε-caprolactone)/gum tragacanth (PCL/GT)	[187]
*Ananas comosus*	bromelain	nanofibers	chitosan	[188]
*Bixa orellana*	bixin	nanofibers	polycaprolactone (PCL)	[189]
*Medicago sativa*	genistein	nanofibers	polycaprolactone (PCL)	[190]
*Thymus vulgaris*	thymol and carvacrol (phenolic monoterpenes)	nanofibers	poly(*ε*-caprolactone) (PCL) and poly(lactic acid) (PLA)	[191]
*Tecomella undulate*	tecomin	nanofibers	polycaprolactone (PCL) and polyvinyl pyrrolidone (PVP)	[192]
*Syzygium aromaticum*	eugenol	magnetic nanospheres	polylactic acid and chitosan	[193]
*Drosera binata*	naphthoquinones (plumbagin)	nanoparticles	silver	[161]
*Centella Asiatica*	pentacyclic triterpenes (*asiatic acid*)	hydrogel, nanoparticles	gelatin, hyaluronic acid, chondroitin sulfate, zinc oxide and copper oxide	[194]
*Scutellaria baicalensis*	baicalin	nanohydrogel	cholesterol	[195]
*Azadirachta indica*	neem oil	liposomes hyalurosomes	argan	[196]
*Mangifera indica*	mangiferin	transferosomes	propylene glycol and glycerol	[197]

**Table 4:** Commercially produced skin substitutes [18,225,226,227,228,229].

Product	Company	Components	Type
Celaderm^TM^	Celadon Science LLC, Brookline, MA, USA	keratinocyte sheets from foreskin	dermal/epidermal, allogeneic
PoliActive^®^	HC Implants BV, Leiden, Netherlands	PEO (polyethylene oxide terephthalate) and PBT (polybuthylene terephthalate) in a porous matrix with cultured keratinocytes and fibroblasts	epidermal, biosynthetic, autogenous
Autoderm (Autologous Inferior Dermal Sling), TransDerm, Lyphoderm, Cryoceal	XCELLentis NV, Gent, Belgium	cultured keratinocytes	epidermal, autogenous
Laserskin, VivoDerm™	Fidia Advanced Biopolymers, Aban Terme, Italy; ER Sąuibb and Sons Inc., Princeton, NJ, USA	a patient’s skin section used for in vitro culture of keratinocytes, which are seeded onto a membrane of hardened, esterified hyaluronic acid perforated with a laser	epidermal, autogenous
Epicel^®^	Genzyme Biosurgery Cambridge, MA, USA	keratinocyte-based cultured epidermal autograft	epidermal, autogenous
EpiDex^®^	Modex Therapeutiques, Lausanne, Switzerland		
EPIBASE	Laboratoires Genevrier, Nice, France		
Permacol^®^, Strattice^®^, Xenoderm^®^	Strattice™ Reconstructive Tissue Matrix (LifeCell Corporation, Branchburg, NJ, USA), MBP (Medical Biomaterial Products, Germany), Tissue Science Laboratories PLC, Aldershot, UK	a porcine-derived collagen matrix and its constituent elastin fibers	dermal, acellular, xenogeneic
Geistlich Derma-Gide™	Geistlich Pharma North America Inc., Princeton, NJ, USA	a porcine, porous, resorbable, 3D matrix	dermal, acellular, xenogeneic
Helicoll™	EnColl Corp., Fremont, CA, USA	a collagen matrix derived from bovine sources	dermal, acellular, xenogeneic
MatriStem, MicroMatrix^®^	ACell Inc., Columbia, MD, USA	matrices derived from the porcine urinary bladder matrix	dermal, acellular, xenogeneic
E-Z-Derm™	Brennen Medical Inc., Saint Paul, Minnesota, USA	a collagen matrix made of porcine collagen cross-linked with aldehyde	dermal, acellular, xenogeneic
Architect^®^ stabilized Collagen matrix	Harbor MedTech, Inc., Irvine, CA, USA.	decellularized equine pericardial tissue	dermal, acellular, xenogeneic
PriMatrix^®^ Dermal Repair Scaffold	TEI Biosciences Inc, Boston, MA, USA	acellular dermal tissue matrix from fetal bovine dermis, rich in type II collagen	dermal, acellular, xenogeneic
Cytal^®^ wound matrix	Acell, Inc., Columbia, MD, USA	porcine urinary bladder matrix with an intact epithelial basement membrane	dermal, acellular, xenogeneic
Matriderm^®^	Skin and Health Care AG, Billerbeck, Germany	a multiporous membrane of bovine origin, composed of collagen (types I, III and V) and a hydrolysate of elastin-alpha, treated with gamma rays	dermal, xenogeneic
Endoform^®^ Natural Dermal Template	Hollister Wound Care, Libertyville, IL, USA	decellularized tissue extracellular matrix derived from ovine forestomach tissue (e.g., collagen I, III, IV, fibronectin, laminin, elastin, hyaluronic acid, heparin sulfate, GAGs, growth factors and chemokines)	dermal, acellular, xenogeneic
Oasis^®^ Wound Matrix	Healthpoint Biotherapeutics, USA; Cook Biotech, Inc., West Lafayette, IN, USA	submucosa of the small intestine of pigs	dermal/epidermal xenogeneic, acellular
Myriad Matrix^®^	Aroa Biosurgery, Auckland, New Zealand	a collagen matrix derived from ovine forestomach	dermal, acellular, xenogeneic
Terudermis^®^	Olympus Terumo Biomaterials Corp., Tokyo, Japan	obtained from heat-denatured bovine collagen, then coated with a silicone film	dermal, xenogeneic
Hyalomatrix^®^ Hyalograft 3D^®^, Hyalomatrix PA^®^	Anika Therapeutics, former Fidia Advanced Biopolymers, Padua, Italy	a bilayer, hyaluronic acid esterified with a benzyl alcohol (Hyaff) matrix or scaffold with an outer silicone membrane	dermal, synthetic
Pelnac Standard Type	Medical Materials Center, Kyoto, Japan	porcine collagen sponge covered with a silicone film	dermal, autologous
STRATAGRAFT	Stratatech Corporation, Madison, WI, USA	allogeneic cultured keratinocytes and dermal fibroblasts in murine collagen-dsat	dermal/epidermal allogeneic
Transcyte^®^	Smith & Nephew, Inc., Largo, Florida, USA	neonatal allogeneic fibroblasts (removed by freezing after producing extracellular matrix and growth factors) cultured and multiplied on nylon fibers and placed on a silicone foil	dermal, allogeneic
Recell	Avita, Northridge, CA, USA	cell suspension of keratinocytes, fibroblasts, Langerhans cells and melanocytes	dermal/epidermal, autologous
OrCei™	Ortec International, Inc., New York, NY, USA	allogeneic fibroblasts and keratinocytes expanded in vitro and seeded on both sides of a bilayer bovine collagen matrix	dermal/epidermal allogeneic
Apligraf	Organogenesis Inc., Canton, Massachusetts, CA, USA; Novartis Pharmaceuticals Corp. East Hanover, NJ, USA	a bilayer composed of a bovine type I collagen lattice with a dermal layer of human fibroblasts and a layer formed from human keratinocytes from cultured cells, from newborn foreskins	dermal/epidermal, cellular, allogeneic and xenogeneic
OrCel	Ortec International, Inc., New York, NY, USA; Forticell Bioscience Inc., NY, USA,	composite allograft synthesized by culturing allogeneic neonatal keratinocytes and fibroblasts in a type I bovine collagen porous sponge with nonporous sides	dermal/epidermal allogeneic
Tissue Tech Autograft System	Fidia Advanced Biopolymers, Aba-no Terre, Italy	membrane made of hyaluronic acid, enriched with cultured autologous fibroblasts and keratinocytes (Hyalograft 3D—a substitute for the dermis, Laserskin—a substitute for the epidermis)	dermal/epidermal autologous
Dermal Regenerative Template DRT, Integra^®^, Integra Bilayer Matrix Wound Dressing, Integra Omnigraft Regeneration Template	Integra LifeSciences Plainsboro, NJ, USA	a bilayer matrix comprising a dermal layer composed of an acellular matrix consisting of cross-linked bovine collagen and chondroitin-6-sulfate, a type of glycosaminoglycan and an overlying silicone layer acting as the epidermis	dermal/epidermal, natural and synthetic, acellular
Dermagraft^®^	Advanced BioHealing, La Jolla, CA, USA; Organogenesis, Canton, MA, USA	a bio-absorbable polyglactin (vicryl) mesh seeded with cryo-preserved neonatal allogeneic foreskin fibroblasts	dermal, cellular, natural and synthetic, biodegradable, allogeneic
Biobrane^®^	Dow Hickam Pharmaceuticals Inc., Sugar Land, TX, USA; Smith & Nephew, London, UK; Mylan Bertek Pharmaceuticals, Durham, North Carolina, USA	biocomposite dressing; nylon mesh with the addition of porcine collagen and a layer of silicone	dermal/epidermal, acellular
TRANSCYTE^®^ Dermagraft-TC ^®^	Advanced Tissue Sciences Inc. (ATS, La Jolla, CA, USA)	a semi-permeable silicone membrane and an extracellular matrix of newborn human dermal fibroblasts cultured on a porcine collagen-coated nylon mesh	dermal, allogeneic
Suprathel^®^	Polymedics Innovations GmbH, Denkendorf, Germany	polylactide copolymer, trimethylene carbonate and ε-caprolactone (Lacto-capromer)	synthetic, biodegradable
NovoSorb^®^ BTM (Biodegradable Temporizing Matrix)	PolyNovo Biomaterials Pty Ltd., Melbourne, Australia	polyurethane bilayer dermal template consisting of a temporary sealing membrane bonded to a 2 mm bioabsorbable open cell matrix	synthetic, biodegradable
Restrata^TM^	Acera Surgical, Inc., St. Louis, MO, USA	electrospun nanofiber matrix	dermal, acellular, synthetic
Alloderm^®^	LifeCell Corporation, Bridgewater, NJ, USA	human cadaver skin that has been chemically treated to remove all cellular material in the dermis	dermal/epidermal, acellular, allogeneic
GraftJacket^®^, GraftJacket RTM	KCI, San Antonio, TX, USA; Wright Medical Group N.V., Memphis, TN, USA	produced from allograft skin	dermal, acellular, allogeneic
GLYADERM ^®^	Euro Skin Bank in the Netherlands	glycerol-preserved acellular dermis	dermal, acellular, allogeneic
Karoderm^®^, SureDerm^®^	Karocell Tissue Engineering AB, Stockholm, Sweden, Hans Biomed Corp., Seoul, Korea, Wright Medical Technology Inc., Arlington, TN, USA	human skin matrix with preserved basement membrane	dermal, acellular, allogeneic
Matrix HD Allograft	RTI Surgical, Alachua, FL, USA	human allograft sterilized using the Tutoplast^®^ Tissue Sterilization process	dermal, acellular, allogeneic
Alloskin, AlloSkin RT™, Alloskin™ AC	AlloSource, Centennial, CO, USA	a fresh-frozen, a fresh irradiated (not frozen), or an acellular meshed dermis-only human skin allograft matrix made from cadaveric tissue, extracellular matrix proteins, glycosaminoglycans and cytokines	dermal, acellular, allogeneic
TheraSkin^®^	LifeNet Health, Virginia Beach, VA, USA	biologically active, cryopreserved human skin	dermal/epidermal, cellular, allogeneic
SkinTE	PolarityTE, Salt Lake City, UT, USA	donated human dermis or autologous skin sample	dermal/epidermal, cellular
DermACELL	LifeNet Health, Virginia Beach, VA, USA	human-derived matrices, sterilized and decellularized to remove immunogenic cellular material	dermal, acellular, allogeneic
AlloPatch^®^, AlloPatch Pliable	Musculoskeletal Transplant Foundation Sports Medicine, Edison, NJ, USA	an open-structure human reticular dermal matrix	dermal, acellular, allogeneic
Dermapure^®^	Tissue Regenix Group, San Antonio, TX, USA	decellularized human dermis	dermal, acellular, allogeneic
Grafix, GrafixPL Prime	Osiris Therapeutics, Inc., Columbia, MD, USA	human amnion chorion membrane	dermal, cellular, allogeneic
WoundEx^®^ Membrane, WoundEx Flow	Skye Biologics, Inc., El Segundo, CA, USA	a dehydrated amniotic membrane, or a flowable human placental connective tissue matrix	dermal, acellular, allogeneic
Xwrap^®^ Amniotic Membrane Derived Allograft	Applied Biologics, Scottsdale, AZ, USA	a chorion-free amniotic membrane wrap, cover, or patch.	dermal, acellular, allogeneic
Amnioexcel^®^	Integra LifeSciences Corp. acquired Derma Sciences, Plainsboro, NJ, USA	a dehydrated human amnion-derived tissue allograft with an intact extracellular matrix	dermal, acellular, allogeneic
Amniomatrix Human Amniotic Suspension Allograft, BioDFactor Viable Tissue Matrix, Biodfence, Integra BioFix Amniotic Membrane Allograft, Integra BioFix Flow Placental Tissue Matrix Allograft	Integra LifeSciences Corp., Plainsboro, NJ, USA; Integra LifeSciences, originally BioD, LLC, Plainsboro, NJ, USA	matrices derived from human placental membrane	dermal, acellular, allogeneic
EpiFix	MiMedx Group Inc., Marietta, GA	the human placental membranes composed of amnion and chorion, rich in extracellular matrix proteins, growth factors and cytokines	dermal, acellular, allogeneic
Affinity^®^ Human Amniotic Allograft	Organogenesis, Inc., Canton, MA, USA	fresh amniotic membrane	dermal, cellular, allogeneic
AmnioRepair	Zimmer Biomet, Warsaw, Poland	a freeze-dried epidermal and dermal replacement with epithelial and stromal sides as well as the outer basement membrane	dermal/epidermal, acellular
Revita^®^	StimLabs, LLC, Roswell, GA, USA	intact human placental membrane	dermal, acellular, allogeneic

## Data Availability

Data sharing not applicable.
